# A Six Years' Trend Analysis of Antimicrobial Resistance Among Bacterial Isolates at Public Health Institute in Amhara Region, Ethiopia

**DOI:** 10.1155/bmri/7676973

**Published:** 2025-01-29

**Authors:** Asrat Agalu Abejew, Teferi Gedif Fenta, Gizachew Yismaw Wubetu

**Affiliations:** ^1^Department of Pharmaceutics and Social Pharmacy, School of Pharmacy, College of Health Sciences, Addis Ababa University, Addis Ababa, Ethiopia; ^2^Department of Pharmacy, College of Medicine and Health Sciences, Bahir Dar University, Bahir Dar, Ethiopia; ^3^Amhara Public Health Institute, Bahir Dar, Ethiopia

**Keywords:** antibiotic resistance, antibiotics, multidrug resistance, trend

## Abstract

**Background:** Antimicrobial resistance (AMR) is one of the top global threats to public health. This study determined trends in AMR from 2016 to 2021 in a regional research laboratory in Northwest Ethiopia.

**Methods:** Data from 2016 to 2021 was extracted from a database. Bacterial identification and resistance tests were made using the standard microbiologic procedures. The results were described, trends in AMR were determined using polynomial regressions, and binary logistic regression at *p* value < 0.05 was used.

**Results:** From 2016 to 2021, 25,143 specimens were sent for culture and susceptibility testing, among which 16,825 (66.9%) bacteria were isolated. About 12,528 (74.5%) isolates were gram-negative, and 4297 (25.5%) were gram-positive. *Klebsiella pneumoniae* (3783, 30.2%) and *Escherichia coli* (3199, 25.5%) were the most common gram-negative bacteria, whereas coagulase-negative *Staphylococcus* spp. (CoNS) (1765, 40.1%) and *Staphylococcus aureus* (1293, 30.1%) were the most common gram-positive bacteria. The overall prevalence of AMR was 2738 (59.9%), of which about 1807 (66.0%) accounted for gram-negative and 931 (34.0%) for gram-positive bacteria. *K. pneumoniae* (743, 80.2%), *Enterobacter cloacae* (196, 74.8%), and *Acinetobacter baumannii* (213, 66.6%) were the most common resistant isolates among gram-negative bacteria, while CoNS (406, 58%), *Streptococcus* species (34, 50%), and *S. aureus* (196, 37%) were from gram-positive bacteria. About 571 (20.9%) of bacteria were resistant to 2–10 drugs. The overall trend of AMR has been rising from year to year, reaching a peak in 2019 which was approximately 66% and then after has been predicted to decline.

**Conclusion:** AMR in the regional laboratory is prevalent and has been increasing although the quadratic equation has revealed downward-opening parabola over time. A growing number of multidrug-resistant bacteria are an alarm to awaken policymakers and those concerned to intervene before it is too late. This calls for a periodic, integrated, and continuing system to monitor AMR for commonly used antibiotics.

## 1. Introduction

During the pre-antibiotic era, infectious diseases were the primary causes of morbidity and mortality [1]. Antibiotic therapy, one of the most significant achievements of humankind in the 20th century, has had a tremendous impact on prolonging life [2]. However, with the increasing rates of antimicrobial resistance (AMR), treatment options are diminishing, and patients are left without viable options if the bacteria become resistant and the available antibiotics no longer work [3]. Although access to antibiotics remains a major issue in many parts of the world, the continued use, overuse, and misuse of antibiotics have accelerated the emergence and spread of resistant bacteria [4]. Furthermore, the number of new classes of antimicrobials entering the market has been drastically decreasing over time. AMR, along with the lack of access to quality antimicrobials, further complicates healthcare [1].

Globally, there are still a substantial number of hospitalizations due to severe bacterial infections [5, 6], resulting in about 7 million deaths in 2019 and accounting for approximately 12% of all global deaths [7]. These deaths were associated with 33 bacterial pathogens, both resistant and susceptible to antimicrobials [8]. AMR has posed a major challenge [9], leading to an estimated 4.95 million deaths associated with bacterial AMR globally in 2019 [10], including 1.05 million deaths in Africa [11]. Additionally, the decrease in new antibiotics entering the market, along with limited access to quality antimicrobials, further complicates healthcare amidst the challenge of AMR [1]. To address the global crisis of AMR, the World Health Organization (WHO) [12] and the US Centers for Disease Control and Prevention (CDC) [13] identified a critical list of AMR “priority pathogens” based on health “threats.”

The COVID-19 pandemic has intensified and complicated AMR [14]. The pandemic has heightened the urgency and risk of AMR due to the overuse of antimicrobials, decreases in routine vaccinations, and disruptions to preventive health services, leading to a rise in untreatable drug-resistant infections and diseases that could potentially trigger another public health emergency [5, 12]. Although it is challenging to estimate the full impact of AMR in low- and middle-income countries (LMICs) [15], AMR poses a particularly severe challenge in these regions, resulting in high mortality and morbidity due to inadequate regulation, limited access to diagnostics, and antimicrobial overprescription [15, 16]. Only 46 (84%) of high-income countries and 58 (61%) of LMICs have developed national AMR action plans (NAPs) [17]. While most African countries have adopted the global action plan as a framework to prevent and combat AMR, fewer countries are able to fully implement country-specific action plans, and numerous challenges persist in many settings [9]. Therefore, despite the significant burden of AMR, substantial gaps remain in the fight against AMR in Africa [9, 11].

In sub-Saharan Africa, several challenges hinder the effective implementation of NAPs [9, 17, 18]. Similarly, in Ethiopia, although the country has committed to partnering globally in the detection and prevention of AMR [9], AMR for many antibiotics and multidrug-resistant (MDR) bacterial strains is becoming increasingly challenging [19, 20]. Furthermore, only a limited number of studies have documented trends in the prevalence of AMR and the antibiotic resistance profiles of bacterial isolates [19, 21]. Thus, this study is aimed at determining AMR trends at the Amhara Public Health Institute (APHI) in Northwest Ethiopia.

## 2. Methods

### 2.1. Study Area, Setting, and Period

Retrospective data was collected based on antimicrobial sensitivity tests conducted from 2016 to 2021 at the APHI Bahir Dar branch. APHI is an autonomous regional public health institute located in Bahir Dar City, one of Ethiopia's top tourist destinations, approximately 578 km from Addis Ababa, the nation's capital. APHI aims to improve health services by generating and delivering scientific evidence, strengthening public health emergency management, and providing quality laboratory services to support effective public health interventions. Overall, the institute serves over 10 million people in Northwest Ethiopia.

### 2.2. Data Collection

Data, including sociodemographic characteristics of patients, isolated bacteria, and antibiotic susceptibility test results, were extracted from a database maintained in the microbiology laboratory unit. Antibiotic susceptibility test results were disaggregated by study year, and trend analysis was conducted based on polynomial equations. In this study, we used the term “MDR” to mean resistance to any drug in any class of at least two antibiotics. Thus, in this study, “MDR” refers to bacterial resistance to more than two antibiotics from any class. Similarly, in this study, “antibiotics” refers to all drugs targeting bacterial pathogens. The antibiotic categories used at APHI include aminoglycosides (AMG), macrolides (Macr), penicillins (Peni), carbapenems (Carb), beta-lactam/beta-lactamase inhibitors (BLIs), cephalosporins (CEF), tetracyclines (TTC), fluoroquinolones (FQs), lincosamides (Linco), sulfamethoxazole-trimethoprim (SXT), nitrofurantoin (NIT), chloramphenicol (CAF), and glycopeptides (Glyc).

### 2.3. Laboratory Diagnostic Methods

The APHI provides laboratory services to the community, including drug susceptibility testing. This study uses data collected from patients seeking culture and sensitivity tests for routine clinical care purposes. Various microbiological specimens—such as blood, urine, stool, swabs, discharges, and body effusions—were used for culture and susceptibility testing. Aseptic techniques were maintained during sample collection, handling, and specimen transport. Standard microbiological procedures were followed for bacterial identification and resistance testing. VITEK 2 methods were primarily used for bacterial isolation, identification, and susceptibility testing, supplemented by manual bacterial culture on different agars and disc diffusion testing with standard antibiotic discs for determining resistance. To verify VITEK 2 results, *Enterococcus casseliflavus* (ATCC 700327), *Kocuria rosea* (ATCC 186), *Staphylococcus aureus* (ATCC 29213), *Staphylococcus lugdunensis* (ATCC 700328), *Staphylococcus sciuri* (ATCC 29061), *Streptococcus pneumoniae* (ATCC 6301), and *Streptococcus uberis* (ATCC 9927) were used for bacterial identification. To ensure the accuracy and reliability of AMR testing, the reference strains *S. aureus* (ATCC 25923), *Escherichia coli* (ATCC 25922), and *Pseudomonas aeruginosa* (ATCC 27853) were used as internal quality controls.

### 2.4. Statistical Analysis

Data were checked for completeness, manually cleaned, entered, and analyzed using SPSS Version 20 statistical software. Descriptive statistics and trend analysis were performed using second-degree polynomial equations. Additionally, binary logistic regression was used to assess associations between sex, age, and type of health facility. A *p* value of < 0.05 was considered statistically significant for determining associations.

### 2.5. Ethics Consideration

Ethical approval was obtained from Addis Ababa University, the College of Health Sciences (protocol code: 106/22/SoP), and the School of Pharmacy (protocol code: ERB/SOP/472/14/2022). The deidentification of records was used to protect patient privacy. All data obtained were kept confidential and used only for this study.

## 3. Results

During the 6 years, 25,143 susceptibility tests were done to determine AMR. The majority of samples were collected from males (13,376, 53.2%) and pediatric patients who were < 15 years old (11,985, 47.6%). The ages of 606 (2.4%) patients were not found to be recorded (Table 1).

### 3.1. Specimen Sources

**Figure 1:**
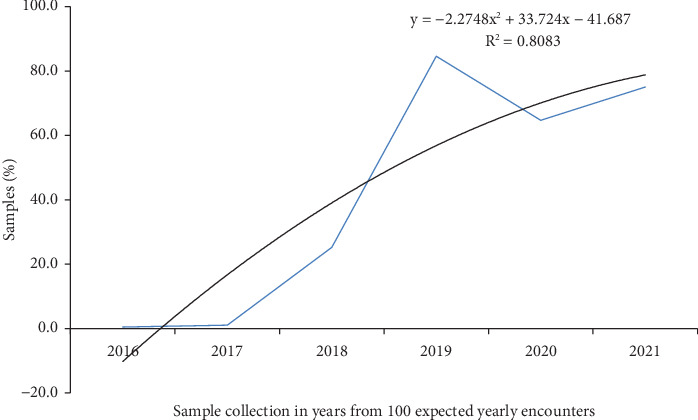
Trends in sample collection (2016–2021), at APHI, Northwest Ethiopia.

The common specimens sent to the APHI for culture and susceptibility testing were blood (11,785, 46.87%), urine (6354, 25.27%), swabs (1993, 7.9%), stool (1620, 6.4%), discharges (436, 1.7%), body effusions (401, 1.6%), and sputum (200, 0.8%). But for 2516 (10%) of the samples, the source was not recorded. The majority of the samples were collected in 2019, 2020, and 2021, with respective values of 8480 (33.7%), 6469 (25.7%), and 7508 (29.9%) (Figure 1).

### 3.2. Bacterial Isolates

The growth of bacteria was evident in 16,825 (66.9%) of the tests, but in 7346 (29.2%), the status of the growth was not recorded, and in 994 (3.9%) of the tests, there was no bacterial growth. The most commonly isolated bacteria were gram-negative bacteria (GNB) (12,528, 74.5%), where *Klebsiella pneumoniae* (3783, 30.2%), *E. coli* (3199, 25.5%), and *Acinetobacter baumannii* (1444, 11.5%) were the most common. On the other hand, gram-positive bacteria (GPB) isolates accounted for 4297 (25.5%), where coagulase-negative *Staphylococcus* spp. (CoNS) (1765, 40.1%), *S. aureus* (1293, 30.1%), and *Enterococcus* spp. (870, 18.9%) were the commonly isolated bacteria (Table 2).

### 3.3. Prevalence of AMR

**Figure 2:**
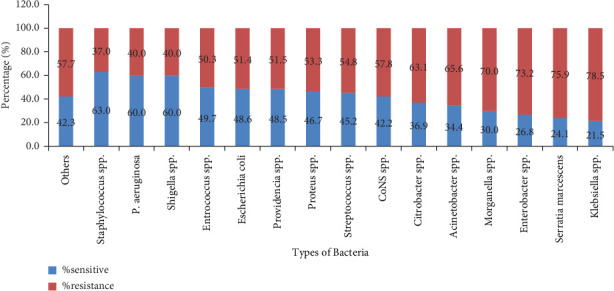
Prevalence of AMR (2016–2021), at APHI, Northwest Ethiopia. Others: *Salmonella choleraesuis*, *Bacillus*, *Moraxella catarrhalis*, and unidentified.

The overall prevalence of AMR was 2738 (59.9%), where about 1807 (66.0%) and 931 (34.0%) of AMR were accounted for GNB and GPB, respectively. *K. pneumoniae* (743, 80.24%), *Enterobacter cloacae* (196, 74.81%), and *A. baumannii* (213, 66.6%) were the most common resistant GNB, whereas CoNS (406, 57.8%), *Streptococcus viridans* (27, 57.8%), and *S. aureus* (196, 36.9%) were the top three resistant GPB. Overall, *Klebsiella* spp. (789, 78.5%), *Serratia marcescens* (22, 75.9%), and *Enterobacter* spp. (257, 73.2%) were highly resistant groups of bacteria (Figure 2).

### 3.4. Antibiotic Resistance Trends

**Figure 3:**
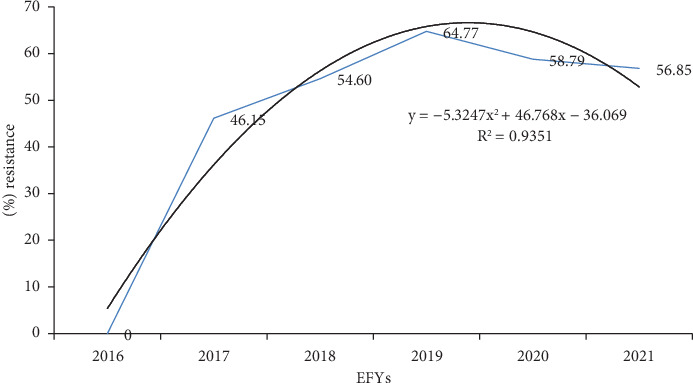
Trends in AMR (2016–2021), at APHI, Northwest Ethiopia.

The AMR trend in a quadratic model exhibits downward-opening parabola with vertex of approximately at (4.4, 66.9) as a function of *y* = −5.3247*x*^2^ + 46.768*x* − 36.069 (*R*^2^ = 0.9351). AMR increased from zero among 81 tested in 2016 to 6579 (64.8%) in 2019, but in 2021, it slightly declined to 2339 (56.9%). In 2017, 2018, and 2020, the AMR was 1512 (46.15%), 3683 (54.6%), and 6356 (58.8%), respectively. Overall, about 93.5% of the variability in the AMR in years can be explained by a second-order polynomial equation (Figure 3).

### 3.5. Antibiotic Resistance Pattern

*A. baumannii* was resistant to ceftriaxone (31, 100%), ciprofloxacin (30, 90.9%), SXT (37, 97.4%), TTC (18, 94.7%), and tobramycin (25, 96.7%). *Enterobacter* spp., *E. coli*, and *K. pneumoniae* were 90%–100% resistant to tobramycin. Although CAF, ceftriaxone, ciprofloxacin, erythromycin, and SXT were the most resistant antibiotics for most bacteria, amikacin, ampicillin/sulbactam, cefepime, cefazolin, and cefoperazone were sensitive to all bacteria (except for *Citrobacter* species (Table 3). Most antibiotics have shown the same trend in resistance from year to year. Ampicillin-resistant *E. coli*, Augmentin-resistant *Acinetobacter*, ceftazidime-resistant *E. coli*, ceftriaxone-resistant *Klebsiella*, and ciprofloxacin-resistant *Klebsiella* increased linearly. The peak resistance was recorded in 2019, and then, it was constant, declining, or rising for most antibiotics (Table 4).

### 3.6. Risk Factors for Resistance

Multivariate binary logistic regression analysis based on variables showed that the age of patients (*p* value = 0.004), gender (*p* value = 0.002), and type of facility (*p* value < 0.01) were strongly associated with the rate of antibiotic resistance. Although the year of the test was strongly associated (*p* value < 0.01), it was found to be confounding when treated with other variables (*p* value = 0.982).

### 3.7. MDR Bacteria

Of 2738 resistant bacteria, 571 (20.9%) were MDR, while 265 (46.2%), 253 (44.2%), and 56 (9.7%) of them were resistant to groups of 2–4, 5–7, and ≥ 8 antibiotics, respectively. The MDR GNB were 378 (18.1%), of which 166 (43.9%), 155 (41.5%), and 55 (14.6%) were resistant to 2–4, 5–7, and ≥ 8 antibiotics, respectively. The most commonly involved GNB in MDR were *K. pneumoniae* (133, 35.2%), *E. coli* (86, 22.8%), *A. baumannii* (39, 10.3%), and *E. cloacae* (37, 9.8%). The MDR among GPB was 193 (20.9%), of which 99 (50.8%), 96 (49.2%), and 1 (0.5%) were resistant to 2–4, 5–7, and ≥ 8 antibiotics, respectively, and CoNS (98, 50%), *S. aureus* (46, 23.6%), and *Enterococcus* spp. (27, 14.2%) were the most common MDR GPB. The group of MDR bacteria with multiple and specific bacteria with resistant antibiotics is presented in Supporting Information 4: Table S1, Supporting Information 5: Table S2, Supporting Information 6: Table S3, and Supporting Information 7: Table S4.

## 4. Discussion

In the past years, infections caused by MDR bacteria have dramatically increased in all parts of the world [22], and among them, resistant *Enterococcus* spp., *S. aureus*, *K. pneumoniae*, *A. baumannii*, *P. aeruginosa*, and *Enterobacter* spp. (ESKAPE) have been increasingly involved in infectious diseases in humans, and antibiotic-resistant ESKAPE bacteria have significantly increased the risk for mortality and healthcare costs in developing countries [9, 11]. Providing evidence-based data on antibiotic resistance, including in livestock and food, and highlighting the need for coordination between animal and human surveillance systems are essential to strengthening infection control measures to reduce the spread of antibiotic-resistant bacteria [12]. Thus, determining the overall and specific trends of AMR has paramount importance.

In this study, the overall prevalence of AMR was 59.9%. It was similar when compared with a systematic review in Ethiopia, which showed an overall prevalence of 59.7% MDR bacteria [19], and in Sierra Leone, among patients with suspected urinary tract infections, the AMR prevalence was 45% [23]. The AMR in the current study was still too high compared with the 29% pooled prevalence of AMR among food handlers [24]. In the current study, the prevalence of AMR GNB was 1807 (66.0%), which was lower compared with the 85.8% MDR GNB referral hospital in Northwest Ethiopia [21]. The difference might be due to differences in geographic and sample types, data collection methods, and the duration of the study, which might account for the variations in resistance patterns across studies.

In this study, the overall trend of antibiotic resistance was linearly increasing over time which had the tendency to decrease during the last two years. It is a fact that AMR is increasing from time to time and poses significant risks to both patients and public health [1]. However, different bacteria were found to have varying rates of resistance to different antibiotics, which may be different from region to region [9]. AMR is a global burden leading to death worldwide, with the highest burdens in low-resource settings [10]; AMR data is unavailable or fragmented in many countries in Africa [11]. Globalization and increased mobility have aided in the spread [25] and increase in AMR worldwide [26]. Although COVID-19 fuels AMR because of increased antibiotic consumption, either by self-medication or by antibiotics prescribed by general practitioners, and reduced antimicrobial stewardship program (ASP) activities [27], the current study showed a decreased trend since COVID-19 which could be justified by a decrease in AMR testing due to shift of attention to contain the pandemic. Understanding the burden of AMR and the leading pathogen–drug combinations contributing to AMR is crucial to making informed and location-specific policy decisions, particularly about infection prevention and control programs, access to essential antibiotics, and the research and development of new vaccines and antibiotics [10].

In this study, *K. pneumoniae* (743, 80.2%), *E. cloacae* (196, 74.8%), and *A. baumannii* (213, 66.6%) were common AMR GNB, whereas CoNS (406, 57.8%), *S. viridans* (27, 57.8%), and *S. aureus* (196, 36.9%) were common GPB with a high AMR rate. This was different from a systematic review in Ethiopia, which reported *E. coli*, *P. aeruginosa*, and *K. pneumoniae* as the most common isolates with high AMR rates [28]. In Indonesia, *K. pneumoniae*, *E. coli*, *P. aeruginosa*, *S. aureus*, and *A. baumannii* were the most common bacteria due to AMR [29]. The common MDR among food handlers, foods, animals, and the environment in Ethiopia were *Staphylococcus* spp. (96%), *Salmonella* spp. (81%), and *E. coli* (77%) [24]. In 2019 alone, resistant *E. coli*, *S. aureus*, *K. pneumoniae*, *S. pneumoniae*, *A. baumannii*, and *P. aeruginosa* were responsible for 3.57 million deaths [10]. Thus, the increasing trend of resistance, geographic differences, and differences in sample types might account for the variations in the resistance patterns across studies.

Resistance to *A. baumannii*, and more importantly, Carb resistance, is one of the top priorities listed as critical by the WHO [13], and it was highly increased (70.3%) during the COVID-19 pandemic [26]. Accordingly, in the present study, imipenem-resistant *A. baumannii* accounts for 33.3%. A systematic review in Indonesia reported 70.7% Carb-resistant *A. baumannii* [29], which is very high. In this study, *A. baumannii* was resistant to ceftriaxone (31, 100%), SXT (37, 97.4%), tobramycin (25, 96.2%), TTC (18, 94.8%), ciprofloxacin (30, 90.9%), gentamicin (28, 82.4%), and ceftazidime (23, 62.3%). Except for the comparable report for ceftazidime (65%), there is high *Acinetobacter* resistance compared with ceftriaxone (73%), SXT (67%), ciprofloxacin (61%), gentamicin (61%), and TTC (56%) [28]. It is also different from a report in Indonesia on *A. baumannii* resistance against gentamicin (67.9%), amikacin (31.0%), tigecycline (30.4%), and colistin (47.5%) [29]. If there is no intervention to tackle the silent tsunami of AMR, *A. baumannii*, which was responsible for more than 250,000 deaths globally in 2019 alone [10], will be one of the future tragedies in the healthcare system of the country. The emergence and spread of *Acinetobacter* species, which are resistant to most available antibiotics, are areas of great concern.

Similarly, in this study, *K. pneumoniae* was resistant to ampicillin (38, 100%), amoxicillin/clavulanic acid (73, 92.4%), ceftriaxone (95, 100%), ciprofloxacin (91, 100%), SXT (10, 100%), gentamicin (115, 95%), TTC (53, 96.4%), tobramycin (82, 91.1%), and ceftazidime (36, 43.4%). Although there are other highly resistant antibiotics, except for ceftazidime (57%), resistance to SXT (66%), amoxicillin/clavulanic acid (61%), tobramycin (60%), TTC (59%), ceftriaxone (56%), gentamicin (47%), and ciprofloxacin (37%) was high in the current study compared with a report in Ethiopia [28]. In Indonesia, low levels of *K. pneumoniae* resistance to 3GC (74.4%), gentamicin (57.3%), SXT (57.3%), and FQs (53.1%) were reported although resistance to colistin (88.5%), cefepime (68.9%), and fosfomycin (47.4%) [29] was different from the report in this study. It is a known fact that antibiotic resistance varies by location and is driven by several factors, such as antibiotic use and infection control practices in healthcare facilities, the underlying health and age of the patient population, and regional spread from nearby locations.

Antibiotic-resistant *P. aeruginosa* is the WHO's top priority [13]. In this study, *P. aeruginosa* showed resistance to ceftazidime (13, 72.2%), ciprofloxacin (15, 75%), gentamicin (9, 100%), and tobramycin (5, 83.3%). This was different from a report in Ethiopia on *P*. *aeruginosa* resistance to TTC (46%–100%), ampicillin (86%–100%), amoxicillin (77%–100%), and SXT (19%–100%) in the environment [24]. It was also low compared with another review in Ethiopia, which reported *P. aeruginosa* resistance to ceftazidime (34%), ciprofloxacin (20%), gentamicin (27%), and tobramycin (5, 83.3%) [28]. This study also revealed a 100% sensitivity of *P. aeruginosa* to cefepime and piperacillin-tazobactam (3, 100%). A 2021 review in Ethiopia reported high *P. aeruginosa* resistance to cefepime (56%) and piperacillin-tazobactam (33%) [28], which was more resistant than that in the current study. In Qatar, high resistance rates of *P. aeruginosa* to cefepime (96.6%), piperacillin/tazobactam (90.7%), and ciprofloxacin (91.2%) were reported, but low resistance rates to gentamicin (73.2%) and tobramycin (54.6%) were reported [22]. The difference might be attributed to differences in the availability and prior consumption of antibiotics [30].

The current study showed 931 (54%) of resistance among GPB, whereas resistant CoNS is 406 (57.8%), *S. viridans* is 27 (57.8%), and *S. aureus* is 196 (36.9%). *S. aureus*, *S. pneumoniae*, and *Streptococcus pyogenes* were the commonly isolated GPB in Ethiopia [26], and in Indonesia, *S. aureus* and *S. pneumonia* [29] were the commonly isolated GPB. *S. aureus* and S. *pneumonia* were among the five leading pathogens, leading to 54.9% of global deaths [8]; methicillin-resistant *Staphylococcus aureus* (MRSA) led to 23% deaths in the WHO African region [11]. In this study, *S. aureus* was resistant to SXT (39, 100%), erythromycin (41, 100%), gentamicin (20, 100%), TTC (29, 93.5%), oxacillin (10, 100%), and clindamycin (9, 100%). Although the overall prevalence of resistance to *S. aureus* is slightly higher than 26.2% in a review in Ethiopia [19], it was lower than the report from Sierra Leone (44%) [23]; resistance to specific antibiotics was different—ceftriaxone (35.3%), ciprofloxacin (20.5%), and norfloxacin (43.5%) in 2019 [19]. It was highly resistant to SXT (44%), erythromycin (45%), gentamicin (29%), TTC (53%), and oxacillin (10, 100%) and clindamycin, compared with a review in Ethiopia [28]. It was high compared with *S. aureus* resistance to erythromycin (28.6%), methicillin (22.2%), and clindamycin (22.0%), except for vancomycin (5.0%) in Indonesia [29]. CoNS were 100% resistant to erythromycin, imipenem, oxacillin, and TTC and 89.1% to ciprofloxacin. This was high compared to CoNS resistance to erythromycin (39%), oxacillin (35%), TTC (47%), and ciprofloxacin (21%) in Ethiopia [28].

In this study, overall 571 (20.9%) cases of MDR were reported, which is comparable with 26.8% in Nepal [6], but it was low compared with the pooled prevalence of MDR from a systematic review in Ethiopia, which documented 59.7% in 2019 [18] and 70.5% in 2021 [20]. The current study also revealed 378 (18.1%) of MDR among GNB, which is lower compared with 85.8% in one of the hospitals in the study area [21] and 74% of MDR among food handlers, in foods, in animals, and in the environment in Ethiopia [24]. The high number of MDR among food handlers, foods, animals, and the environment [24] confirmed the complexity of socioecologic systems that drive the emergence and spread of AMR [30]. The potential crisis caused by MDR and the limited treatment options could be solved by integrating the pharmaceutical industry, research institutions, and other stakeholders [31]. There should be efforts at the health facility level to improve antibiotic prescribing, and isolated pathogen management will play a major role in controlling the spread of organisms [32]. This calls for effective policy and intervention measures to address AMR through the One Health approach.

Overall, resistant *E. coli*, *S. aureus*, *K. pneumoniae*, *S. pneumoniae*, *A. baumannii*, and *P. aeruginosa* were the leading causes of death [10]. AMR, a multifaceted problem that affects almost all communities [1], is a complex process involving multiple factors beyond antibiotics [33, 34]. It requires coordinated strategies and actions from various stakeholders to curtail the emergence and spread of AMR [1]. Continuous monitoring, molecular detection of the gene responsible for AMR, and the implementation of recommended ASPs are important to reduce the emergence and spread of drug-resistant pathogens [2, 21]. While antibiograms are helpful, they cannot be relied upon as the sole resource for guiding empirical antimicrobial therapy since the patient's microbiological history and prior antibiotic use may provide more helpful information during therapy selection [35]. Thus, implementing context-specific and locally tailored ASPs in healthcare facilities is highly recommended to reduce inappropriate antibiotic use and resistance [2, 35].

The current study will serve an alarm to awaken health professionals during disease management and monitoring of treatment. It will also help in addressing AMR to safeguard public health and inform effective interventions. However, the current study uses data which was not systematically collected from the population; it will not show an accurate picture of AMR in the area. However, it gives us a clue on the past trend among those routinely tested to predict the likelihood of an AMR occurrence in the future. Another limitation is that it may not include all antibiotics currently in use. A significant number of the tests were incomplete, which may understate the real picture of all tests done in the laboratory.

In conclusion, AMR is prevalent in the Amhara public health research laboratory. It has been increasing, reaching a peak in 2019, and started to decline in 2020 and will possibly decline over time. The vast majority of antibiotic-resistant and MDR bacteria were GNB. The decline trend in AMR and sample collection in the laboratory clearly indicated the impact of COVID-19 on the AMR surveillance. This calls for sustainable programs for surveillance and monitoring of AMR in the region. Overall, implementation of context-specific ASPs will be a cost-effective strategy to contain and delay the spread of the AMR tsunami.

## Figures and Tables

**Table 1:** Distribution of samples by different variables (2016–2021), at APHI, Northwest Ethiopia (*N* = 25,143).

**Variables**	**Frequency (%)**
Sex	
Male	13,376 (53.2)
Female	11,767 (46.8)
Age	
< 15 years	11,985 (47.6)
15–64 years	11,205 (44.6)
≥ 65 years	1347 (5.4)
Not recorded	606 (2.4)
Facility	
Public facility	19,194 (76.3)
Private facility	4867 (19.4)
Not known	1083 (4.3)
Total	25,143 (100)
Residence	
Bahir Dar City	23,719 (98.6)
Awi Zone	176 (0.7)
South Gondar Zone	111 (0.5)
West Gojjam Zone	50 (0.2)
Gondar City	4 (0.02)
Not specified	1083 (4.3)
Total	25,143 (100)

**Table 2:** Distribution of commonly isolated bacteria (2016–2021), at APHI, Northwest Ethiopia (*N* = 16,825).

**Type of bacteria**	**Isolated specific bacteria**	**Frequency (%)**
Gram-negative bacteria	*Klebsiella pneumoniae*	3783 (30.2)
	*Escherichia coli*	3199 (25.5)
	*Acinetobacter baumannii*	1444 (11.5)
	*Enterobacter* species	1330 (10.6)
	*Pseudomonas aeruginosa*	891 (7.1)
	*Klebsiella* species	615 (4.9)
	*Citrobacter* species	470 (3.8)
	*Proteus species*	216 (1.7)
	*Serratia marcescens*	135 (1.1)
	*Providencia* species	135 (1.1)
	*Shigella* species	102 (0.8)
	*Acinetobacter* species	77 (0.6)
	Unspecified	131 (1.1)
	Total gram-negative	12,528 (74.5)

Gram-positive bacteria	CoNS	1765 (40.1)
	*Staphylococcus aureus*	1293 (30.1)
	*Enterococcus* species	870 (18.9)
	*Streptococcus viridans*	213 (5.0)
	*Streptococcus* species	73 (1.7)
	*Staphylococcus* species	48 (1.1)
	Unspecified	35 (0.8)
	Total gram-positive bacteria	4297 (25.5)

Total isolates		16,825 (100)

**Table 3:** Antibiotic resistance pattern (2016–2021), at APHI, Northwest Ethiopia.

**Antibiotic**	***A. baumannii***	***Acinetobacter* species**	***Citrobacter* species**	**CoNS species**	***Enterobacter aerogenes***	***Enterobacter cloacae***	***Enterococcus faecalis***	***Enterococcus* species**	***E. coli***	***K. oxytoca***	***K. ozaenae***	***K. pneumoniae***	***K. rhinoscleromatis***	***Morganella* species**	***Proteus mirabilis***	***Proteus vulgaris***	***Providencia* species**	***P. aeruginosa***	***Serratia marcescens***	***Shigella* species**	***S. aureus***	***Staphylococcus* species**	***Streptococcus* species**	***S. viridans***	**Others** ^**a**^	**Total ** **R** ** (%)**
	**R** ** (%)**	**R** ** (%)**	**R** ** (%)**	**R** ** (%)**	**R** ** (%)**	**R** ** (%)**	**R** ** (%)**	**R** ** (%)**	**R** ** (%)**	**R** ** (%)**	**R** ** (%)**	**R** ** (%)**	**R** ** (%)**	**R** ** (%)**	**R** ** (%)**	**R** ** (%)**	**R** ** (%)**	**R** ** (%)**	**R** ** (%)**	**R** ** (%)**	**R** ** (%)**	**R** ** (%)**	**R** ** (%)**	**R** ** (%)**	**R** ** (%)**	**R** ** (%)**
Amoxicillin				0/28				1 (33.3)													1 (3.3)	0/3				2 (3.125)
Amikacin	0/5		0/2		0/1	0/4			0/16			0/3		0/2				0/13							0/2	0/48
Ampicillin			7 (100)	0/17	2 (100)	12 (100)	1 (100)	17 (77.3)	29 (100)	2 (100)	3 (100)	38 (100)	1 (100)	1 (100)	3 (100)		1 (100)		1 (100)		1 (5)					119 (74.4)
Amp/sulbactam	0/5		0/3		0/3	0/6			0/31			0/3		0/1	0/2											0/54
Augmentin	4 (80)		11 (78.6)		11 (84.6)	22 (95.7)			49 (92.5)	8 (100)	1 (50)	73 (92.4)	1 (100)	1 (100)	3 (75)	1 (100)	2 (100)		1 (100)							188 (90.8)
Azithromycin				3 (17.6)				0/2													1 (3.8)	0/3				4 (8.3)
Cefepime	0/22		4 (57.1)		0/9	0/10			0/41	0/8	0/6	0/44			0/3	0/2	0/5	0/18		0/1						4 (7.7)
Cefazolin	0/11	0/1	2 (66.7)		0/2	0/5			0/28		0/3	0/11			0/3	0/1		0/1	0/1	0/1						2 (4)
Cefoperazone									0/1																	0/48
Cefoxitin	0/19		0/7	7 (100)	0/3	2 (20)		0/3	1 (1.3)		0/6	2 (8.3)			1 (14.3)	0/1	0/4	0/22	0/2		3 (100)					16 (8.2)
Ceftazidime	23 (62.2)	0/1	4 (80)		1 (100)	5 (29.4)			20 (48.9)	6 (66.7)	1 (50)	36 (43.4)	0/1	0/1	1 (100)	2 (100)		13 (72.2)	1 (33.3)	0/1						113 (50.7)
Ceftriaxone	31 (100)	1 (100)	6 (85.7)		10 (100)	21 (100)			39 (92.9)	10 (100)	5 (83.3)	95 (100)		1 (100)	3 (100)	2 (100)	2 (66.7)	1 (100)	2 (100)						1 (100)	230 (97.5)
Cefuroxime			0/3		0/3	0/3			0/64		0/2	0/6		0/1		0/1	0/1	0/1			1 (100)				1 (100)	2 (2.3)
Chloramphenicol			2 (100)	22 (75.9)		7 (100)	1 (100)	8 (38.1)	3 (100)		1 (100)	7 (100)	1 (100)		2 (100)		1 (100)				6 (50)			9 (100)	1 (100)	71 (73.4)
Ciprofloxacin	30 (90.9)	1 (100)	8 (100)	58 (89.2)	4 (100)	25 (100)	2 (100)	3 (75)	64 (100)	2 (100)	5 (100)	91 (100)	1 (100)	2 (100)	3 (100)			15 (75)	5 (100)	0/2	31 (64.6)	1 (33.3)			4 (100)	355 (90.3)
Clindamycin				30 (100)				1 (6.7)													9 (100)		0/1	5 (83.3)		45 (73.8)
SXT	37 (97.4)		10 (100)	88 (100)	10 (100)	30 (100)		1 (100)	79 (100)	10 (100)	8 (100)	125 (100)	1 (100)	3 (100)	3 (100)	1 (100)	5 (100)	3 (60)	4 (100)	1 (100)	39 (100)	3 (100)	1 (100)		5 (100)	467 (99.4)
Doxycycline									0/1																	0/1
Erythromycin				84 (100)			3 (100)	30 (100)						1 (100)							41 (100)	2 (100)	3 (100)	13 (100)		177 (100)
Gentamicin	28 (82.4)	1 (100)	7 (87.5)	34 (100)	9 (90)	27 (79.4)			29 (72.5)	8 (66.7)	5 (71.4)	115 (95.0)	1 (100)	1 (100)	0/1	1 (100)	1 (50)	9 (100)	3 (100)		20 (100)				1 (100)	301 (88.3)
Imipenem	9 (33.3)		2 (33.3)		2 (40)	7 (53.8)			0/45	0/1	2 (33.3)	8 (36.4)			0/3	0/1	0/3	3 (15)	0/1							33 (21.6)
Nitrofurantoin	4 (100)		4 (100)			4 (100)		1 (100)	11 (78.6)			6 (100)			3 (100)		1 (50)	2 (100)	2 (100)		0/1					38 (88.4)
Oxacillin				24 (100)																	24 (100)					34 (100)
Penicillin				1 (100)				15 (93.8)													1 (100)		1 (100)			18 (94.7)
Tetracycline	18 (94.7)	1 (100)	9 (100)	50 (96.2)	7 (100)	9 (100)	2 (100)	7 (63.6)	41 (100)	3 (100)	4 (100)	53 (96.4)	1 (100)	2 (100)	3 (100)		2 (100)	1 (100)	1 (100)		29 (93.5)	1 (100)	1 (100)	0/1	1 (50)	246 (95)
Tobramycin	25 (96.2)	1 (100)	5 (100)		0/1	22 (100)			33 (97.1)	6 (100)	2 (66.7)	82 (91.1)	1 (100)	1 (100)			1 (100)	5 (83.3)	2 (66.7)						1 (100)	187 (93.0)
Others^b^	3 (100)		1 (100)	0/1		0/1		5 (83.3)	3 (100)									4 (100)			0					16 (80)
No antibiotic		3 (30)	1 (10)	5 (7.5)	5 (100)	3 (60)	0/1	0/5	30 (49.2)	0/1	1 (33.3)	12 (54.5)								1 (50)	2.3		4 (80)	0/2	3 (27.3)	70 (23.7)
Total	212 (66.5)	8 (47.1)	83 (68.6)	406 (74.6)	61 (68.5)	196 (75.1)	9 (90)	89 (63.6)	431 (53.4)	55 (76.4)	38 (56.7)	743 (80.3)	8 (88.9)	14 (73.7)	25 (56.8)	7 (53.8)	16 (50)	56 (39.7)	22 (75.9)	2 (25)	195 (51.5)	7 (46.7)	10 (83.3)	27 (87.1)	18 (62.1)	2738 (67.2)

Abbreviation: SXT: sulfamethoxazole/trimethoprim.

^a^ *Salmonella choleraesuis* and unspecified gram-positive and gram-negative bacteria.

^b^Meropenem, norfloxacin, piperacillin-tazobactam, and vancomycin.

**Table 4:** Trends in antibiotic-resistant bacteria (2016–2021), at APHI, Northwest Ethiopia.

**Antibiotic-resistant bacteria**	**Years**					
	**2017**	**2018**	**2019**	**2020**	**2021**	**Total**
	**R** ** (%)**	**R** ** (%)**	**R** ** (%)**	**R** ** (%)**	**R** ** (%)**	**R** ** (%)**
Amoxicillin-resistant *Enterococcus*			1 (100)	0/1		1 (50)
Amoxicillin-resistant *S. aureus*	1 (100)					1 (100)
Ampicillin-resistant *Acinetobacter*			1 (100)		6 (85.7)	7 (87.5)
Ampicillin-resistant *E. coli*			3 (75)	7 (87.50	19 (95)	29 (90.6)
Ampicillin-resistant *Enterobacter*			4 (100)	3 (75)	7 (100)	14 (93.3)
Ampicillin-resistant *Enterococcus*			4 (100)	0/3	14 (66.7)	18 (62.1)
Ampicillin-resistant *Klebsiella*			21 (100)	10 (100)	13 (92.9)	44 (97.8)
Ampicillin-resistant *Morganella*			1 (100)			1 (100)
Ampicillin-resistant *Proteus*				2 (66.7)	1 (1000	3 (75)
Ampicillin-resistant *Providencia*					1 (100)	1 (100)
Ampicillin-resistant *S. aureus*			1 (100)			1 (100)
Ampicillin-resistant *Streptococcus*						0/1
Ampicillin-resistant *Serratia*					1 (100)	1 (100)
Augmentin-resistant *Acinetobacter*			3 (75%)	3 (100)	9 (81.8)	15 (83.3)
Augmentin-resistant *E. coli*		1 (50)	8 (72.7)	31 (79.5)	19 (57.6)	59 (78.7)
Augmentin-resistant *Enterobacter*			10 (100)	4 (80)	19 (95)	33 (94.3)
Augmentin-resistant *Klebsiella*			34 (91.9)	27 (93.1)	22 (73.3)	83 (86.5)
Augmentin-resistant *Morganella*			0/1	1 (100)		1 (50)
Augmentin-resistant *Proteus*				1 (33.3)	3 (100)	4 (66.7)
Augmentin-resistant *Providencia*				1 (100)	1 (50)	2 (66.7)
Augmentin-resistant *Serratia*				1 (100)	0/1	1 (50)
Azithromycin-resistant CoNS				3 (75)		3 (75)
Azithromycin-resistant *Enterococcus*			0/1	1 (100)		0/1
Azithromycin-resistant *S. aureus*				1 (100)	0/1	1 (25)
Cefotaxime-resistant *Salmonella*				0/1		0/1
Cefoxitin-resistant CoNS					7 (87.5)	7 (87.5)
Cefoxitin-resistant *E. coli*			1 (100)	0/3		1 (25)
Cefoxitin-resistant *Enterobacter*					2 (100)	2 (100)
Cefoxitin-resistant *Klebsiella*			0/2		2 (100)	2 (50)
Cefoxitin-resistant *Proteus*					1 (100)	1 (100)
Cefoxitin-resistant *S. aureus*					3 (33.3)	3 (33.3)
Ceftazidime-resistant *Acinetobacter*			7 (77.8)	8 (88)	12 (75)	27 (79.4)
Ceftazidime-resistant *E. coli*			3 (30)	9 (69.2)	8 (72.7)	20 (58.8)
Ceftazidime-resistant *Enterobacter*			3 (100)	2 (50)	1 (25)	6 (54.5)
Ceftazidime-resistant *Klebsiella*			17 (85)	21 (100)	5 (100)	43 (93.4)
Ceftazidime-resistant *Morganella*				0/1		0/2
Ceftazidime-resistant *Proteus*				1 (100)	2 (100)	3 (100)
Ceftazidime-resistant *Pseudomonas*			4 (44.4)	2 (0.4)	7 (58.3)	13 (50)
Ceftazidime-resistant *Serratia*				1 (100)		1 (100)
Ceftriaxone-resistant *Acinetobacter*			10 (83.3)	11 (91.7)	18 (78.3)	39 (83)
Ceftriaxone-resistant *E. coli*	1 (100)		2 (22.2)	17 (58.6)	19 (61.3)	39 (55.7)
Ceftriaxone-resistant *Enterobacter*			9 (90)	6 (75)	15 (71.4)	30 (76.9)
Ceftriaxone-resistant *Klebsiella*			43 (95.6)	39 (97.5)	29 (100)	111 (97.4)
Ceftriaxone-resistant *Morganella*			1 (100)	4 (80)		1 (50)
Ceftriaxone-resistant *Proteus*				2 (50)	3 (100)	5 (71.4)
Ceftriaxone-resistant *Providencia*				1 (100)	1 (100)	2 (100)
Ceftriaxone-resistant *Pseudomonas*				1 (100)		1 (100)
Ceftriaxone-resistant *Salmonella*				0/1		0/1
Ceftriaxone-resistant *Serratia*				1 (100)	1 (100)	2 (100)
Cefuroxime-resistant *S. aureus*	1 (100)					1 (100)
Cefuroxime-resistant *Salmonella*				1 (100)		1 (100)
Chloramphenicol-resistant *Acinetobacter*				2 (100)	0/4	2 (33.3)
Chloramphenicol-resistant CoNS			7 (38.9)	9 (32.1)	6 (20.7)	22 (29.3)
Chloramphenicol-resistant *E. coli*			2 (100)	1 (33.3)	0/2	3 (42.9)
Chloramphenicol-resistant *Enterobacter*			1 (100)	3 (75)	3 (60)	7 (70)
Chloramphenicol-resistant *Enterococcus*			3 (60)	5 (38.5)	1 (5.6)	9 (25)
Chloramphenicol-resistant *Klebsiella*			3 (75)	5 (83.3)	2 (28.6)	10 (58.8)
Chloramphenicol-resistant *Proteus*				1 (50)	1 (100)	2 (66.7)
Chloramphenicol-resistant *Providencia*				1 (100)		1 (100)
Chloramphenicol-resistant *S. aureus*			2 (11.1)	2 (10.5)	2 (11.8)	6 (11.1)
Chloramphenicol-resistant *Streptococcus*			8 (57.1)	1 (100)	0/2	9 (52.9)
Ciprofloxacin-resistant *Acinetobacter*			12 (54.5)	12 (75)	15 (50)	39 (57.4)
Ciprofloxacin-resistant CoNS		0/3	11 (47.8)	16 (64)	31 (64.6)	58 (58.6)
Ciprofloxacin-resistant *E. coli*		0/1	20 (54.1)	26 (72)	18 (56.3)	64 (60.4)
Ciprofloxacin-resistant *Enterobacter*			14 (73.7)	4 (50)	11 (52.4)	29 (60.4)
Ciprofloxacin-resistant *Enterococcus*			4 (66.7)	1 (25)	0/2	5 (41.7)
Ciprofloxacin-resistant *Klebsiella*			51 (60.7)	28 (65.1)	21 (65.6)	100 (62.9)
Ciprofloxacin-resistant *Morganella*			1 (100)	1 (100)		2 (100)
Ciprofloxacin-resistant *Proteus*				3 (60)	0/3	3 (37.5)
Ciprofloxacin-resistant *Providencia*			0/2	0/1	0/2	0/5
Ciprofloxacin-resistant *Pseudomonas*			5 (45.5)	3 (50)	7 (43.8)	15 (45.5)
Ciprofloxacin-resistant *S. aureus*	0/1		11 (50)	11 (44)	11 (40.7)	33 (44.6)
Ciprofloxacin-resistant *Salmonella*				1 (100)		1 (100)
Ciprofloxacin-resistant *Shigella*			2 (66.7)	0/1		2 (50)
Ciprofloxacin-resistant *Serratia*			3 (100)	1 (100)	1 (100)	5 (100)
Clindamycin-resistant CoNS		0/3	6 (25)	10 (35.7)	14 (27.5)	30 (28.3)
Clindamycin-resistant *Enterococcus*			1 (100)			1 (100)
Clindamycin-resistant *S. aureus*			4 (18.2)	3 (12)	2 (8)	9 (12.5)
Clindamycin-resistant *Salmonella*				0/1		0/1
Clindamycin-resistant *Streptococcus*			4 (28.6)	1 (100)	0/2	5 (29.4)
Erythromycin-resistant CoNS		2 (66.7)	22 (88)	25 (86.2)	35 (81.4)	84 (84)
Erythromycin-resistant *Enterococcus*			7 (77.8)	12 (100)	14 (82.4)	33 (86.8)
Erythromycin-resistant *S. aureus*	1 (100)		15 (65.2)	12 (48)	15 (62.5)	43 (58.9)
Erythromycin-resistant *Streptococcus*			14 (93.3)	1 (100)	1 (50)	16 (88.9)
Gentamicin-resistant *Acinetobacter*			11 (55)	9 (75)	16 (53.3)	36 (58.1)
Gentamicin-resistant CoNS			4 (21.1)	12 (54.5)	18 (41.9)	34 (40.5)
Gentamicin-resistant *E. coli*	0/1	1 (50)	10 (27.8)	9 (26.5)	9 (27.3)	29 (27.4)
Gentamicin-resistant *Enterobacter*			18 (100)	5 (62.5)	13 (61.9)	36 (76.6)
Gentamicin-resistant *Enterococcus*			0/1		0/1	0/2
Gentamicin-resistant *Klebsiella*			69 (81.2)	38 (92.7)	23 (71.9)	130 (82.3)
Gentamicin-resistant *Morganella*			1 (100)	1 (100)		2 (100)
Gentamicin-resistant *Proteus*				0/5	1 (33.3)	1 (12.5)
Gentamicin-resistant *Providencia*			0/2	0/1	1 (50)	1 (20)
Gentamicin-resistant *Pseudomonas*			4 (36.4)	1 (20)	4 (26.7)	9 (29)
Gentamicin-resistant *S. aureus*			5 (26.3)	6 (15)	9 (34.6)	20 (30.3)
Gentamicin-resistant *Serratia*			3 (100)	0/1	0	3 (60)
Imipenem-resistant *Acinetobacter*			5 (55.6)	3 (37.5)	3 (30)	11 (40.7)
Imipenem-resistant *E. coli*			0/5	0/12	0/4	0/21
Imipenem-resistant *Enterobacter*			6 (50)	0/3	3 (50)	9 (42.9)
Imipenem-resistant *Klebsiella*			3 (7)	6 (40)	1 (25)	10 (16.1)
Imipenem-resistant *Morganella*				0/1		0/1
Imipenem-resistant *Pseudomonas*			2 (66.7)	0/1	1 (25)	3 (37.5)
Imipenem-resistant *Serratia*						0/2
Meropenem-resistant *Klebsiella*					0/1)	0/1
Meropenem-resistant *Acinetobacter*					2 (66.7)	2 (66.7)
Meropenem-resistant *E. coli*				1 (33.3)		1 (33.3)
Meropenem-resistant *Providencia*					0/1	0/1
Nitrofurantoin-resistant *Acinetobacter*			3 (100)	3 (100)	2 (40)	8 (72.7)
Nitrofurantoin-resistant CoNS		0/3	0/1			0/4
Nitrofurantoin-resistant *E. coli*	0/1	1 (33.3)	5 (18.5)	3 (12)	2 (8.3)	11 (13.8)
Nitrofurantoin-resistant *Enterobacter*			1 (33.3)	2 (100)	1 (20)	4 (40)
Nitrofurantoin-resistant *Enterococcus*			0/1	1 (25)	0/2	1 (14.3)
Nitrofurantoin-resistant *Klebsiella*			3 (75)	2 (40)	1 (20)	6 (42.9)
Nitrofurantoin-resistant *Morganella*			0/1			0/1
Nitrofurantoin-resistant *Proteus*				2 (100)	1 (50)	3 (75)
Nitrofurantoin-resistant *Providencia*			2 (100)		0/1	2 (66.7)
Nitrofurantoin-resistant *Pseudomonas*			1 (50)	1 (100)		2 (66.7)
Nitrofurantoin-resistant *S. aureus*	0/1		0/3	0/4	0/2	0/10
Nitrofurantoin-resistant *Serratia*			1 (100)	1 (100)		2 (100)
Norfloxacin-resistant *E. coli*	1 (100)	1 (100)				2 (100)
Oxacillin-resistant CoNS			3 (75)	10 (55.6)	11 (68.8)	24 (63.2)
Oxacillin-resistant *Enterococcus*			0/1			0/1
Oxacillin-resistant *S. aureus*			4 (36.4)	6 (3)	1 (14.3)	11 (28.9)
Penicillin-resistant CoNS			1 (100)			1 (100)
Penicillin-resistant *Enterococcus*					15 (57.7)	15 (48.4)
Penicillin-resistant *S. aureus*			1 (100)			1 (50)
Penicillin-resistant *Salmonella*						0/1
Penicillin-resistant *Streptococcus*			1 (100)			1 (100)
Piperacillin-tazobactam–resistant *Acinetobacter*					3 (50)	3 (50)
Piperacillin-tazobactam–resistant *Pseudomonas*			0/2		4 (44.4)	4 (36.4)
Sulfamethoxazole-resistant *Acinetobacter*			14 (66.7)	15 (100)	18 (69.2)	47 (75.8)
Sulfamethoxazole-resistant CoNS		0/3	21 (87.5)	25 (89.3)	42 (80.8)	88 (82.2)
Sulfamethoxazole-resistant *E. coli*	1 (100)	1 (50)	23 (63.9)	30 (88.2)	24 (60.6)	79 (73.1)
Sulfamethoxazole-resistant *Enterobacter*			18 (94.7)	7 (87.5)	15 (75)	40 (85.1)
Sulfamethoxazole-resistant *Enterococcus*			1 (100)			1 (100)
Sulfamethoxazole-resistant *Klebsiella*			77 (91.7)	41 (95.3)	27 (84.4)	145 (91.2)
Sulfamethoxazole-resistant *Moraxella*			1 (100)			1 (100)
Sulfamethoxazole-resistant *Morganella*			1 (100)	1 (100)		2 (100)
Sulfamethoxazole-resistant *Proteus*				3 (0.6)	1 (33.3)	4 (50)
Sulfamethoxazole-resistant *Providencia*			2 (100)	1 (100)	2 (100)	5 (100)
Sulfamethoxazole-resistant *Pseudomonas*			0/1	3 (100)		3 (75)
Sulfamethoxazole-resistant *S. aureus*			13 (54.2)	15 (53.6)	14 (48.3)	42 (51.8)
Sulfamethoxazole-resistant *Shigella*			1 (100)			1 (50)
Sulfamethoxazole-resistant *Streptococcus*					1 (100)	1 (100)
Sulfamethoxazole-resistant *Serratia*			2 (66.7)	1 (100)	1 (100)	4 (80)
Tetracycline-resistant *Acinetobacter*			4 (100)	10 (90.9)	14 (70)	28 (80)
Tetracycline-resistant CoNS			3 (100)	13 (76.5)	34 (77.3)	50 (78.1)
Tetracycline-resistant *E. coli*		1	5 (100)	17 (73.9)	18 (81.8)	41 (80.4)
Tetracycline-resistant *Enterobacter*			6 (100)	1 (25)	9 (64.3)	16 (66.7)
Tetracycline-resistant *Enterococcus*			5 (100)	3 (75)	1 (100)	9 (90)
Tetracycline-resistant *Klebsiella*			10 (90.1)	30 (83.3)	22 (81.5)	62 (83.8)
Tetracycline-resistant *Morganella*			1 (100)	1 (100)		2 (100)
Tetracycline-resistant *Proteus*				2 (100)	1 (50)	3 (75)
Tetracycline-resistant *Providencia*				1 (100)	1 (50)	2 (66.7)
Tetracycline-resistant *Pseudomonas*				1 (100)		1 (100)
Tetracycline-resistant *S. aureus*	0/1		6 (66.7)	14 (58.3)	10 (47.6)	30 (54.5)
Tetracycline-resistant *Salmonella*						0/1
Tetracycline-resistant *Streptococcus*					1 (100)	1 (100)
Tetracycline-resistant *Serratia*				1 (100)		1 (100)
Tobramycin-resistant *Acinetobacter*			10 (58.8)	8 (57.1)	13 (59.1)	31 (58.5)
Tobramycin-resistant *E. coli*			4 (17.4)	19 (54.3)	10 (50)	33 (42.3)
Tobramycin-resistant *Enterobacter*			10 (71.4)	6 (75)	6 (66.7)	22 (71)
Tobramycin-resistant *Klebsiella*			49 (80.3)	32 (91.4)	11 (73.3)	92 (82.9)
Tobramycin-resistant *Morganella*				1 (100)		1 (100)
Tobramycin-resistant *Proteus*					0/1	0/0
Tobramycin resistant *Providencia*			0/2		1 (100)	1 (25)
Tobramycin-resistant *Pseudomonas*			4 (44.4)		1 (11.1)	5 (22.7)
Tobramycin-resistant *Serratia*			2 (100)			2 (66.6)
Vancomycin-resistant *Enterococcus*				2 (100)	4 (22.2)	6((30)
Vancomycin-resistant *Streptococcus*					0/2	0/2
Total	6 (54.5)	8 (29.6)	885 (62.7)	834 (63)	928 (57.4)	2661 (60.6)

## Data Availability

All data are included in the manuscript. If additional data are required, we will provide them upon request.

## Supporting Information

Supporting Information 1 Table S1: Distribution of MDR gram-positive bacteria (2016–2021), APHI, Amhara Region, Ethiopia (*N* = 195).

Supporting Information 2 Table S2: Distribution of MDR gram-negative bacteria (2016–2021), APHI, Amhara Region, Ethiopia (*N* = 378).

Supporting Information 3 Table S3: Distribution of MDR gram-negative bacteria (2016–2021), APHI, Amhara Region.

Supporting Information 4 Table S4: Distribution of MDR gram-positive bacteria in APHI during the last 6 years, 2016–2021 (*N* = 195).
