# Supplementary material for: A Six Years' Trend Analysis of Antimicrobial Resistance Among Bacterial Isolates at Public Health Institute in Amhara Region, Ethiopia
Source: Biomed Res Int. 2025 Jan 29;2025:7676973. doi: 10.1155/bmri/7676973 (PMC11824853; doi:10.1155/bmri/7676973)
Supplement: Supporting Information 1 — Table S1: Distribution of MDR gram-positive bacteria (2016–2021), APHI, Amhara Region, Ethiopia (N = 195). [file 7676973.f1.docx]

Supplementary Table 1: Distribution of MDR gram-positive bacteria (2016-2021), at APHI, Northwest Ethiopia (N=195)

| Class of Antibiotics | Type of Bacterial isolates | | | |
| --- | --- | --- | --- | --- |
|  | Enterococcus | Streptococcus | CoNS | S aureus |
| CAF, Macr | 5 | 5 | 1 |  |
| SXT, Macr |  |  | 7 | 3 |
| Peni, Macr | 5 |  |  | 3 |
| Peni2 | 5 |  |  |  |
| FQ., SXT |  |  | 1 | 2 |
| FQ, Macr |  |  | 2 | 1 |
| SXT, TTC |  |  | 2 | 1 |
| FQ, TTC | 1 |  |  | 1 |
| Macr, TTC |  |  |  | 2 |
| Macr, Glyc | 2 |  |  |  |
| Others* |  |  |  | 3 |
| SXT, Macr, TTC |  | 1 | 1 | 2 |
| Peni2, Macr | 3 |  |  |  |
| Macr2, SXT |  |  | 2 | 1 |
| CAF, Linco, Macr |  | 3 |  |  |
| FQ, SXT, AMG |  |  | 2 | 1 |
| FQ, SXT, TTC |  |  | 2 | 1 |
| Linco, SXT, Macr |  |  | 3 |  |
| FQ., SXT, Macr |  |  | 3 | 1 |
| Peni, CAF, Macr | 2 |  |  |  |
| FQ, SXT, Macr |  |  | 1 | 1 |
| FQ, Macr, TTC | 2 |  |  |  |
| SXT, Macr, AMG |  |  | 1 | 1 |
| Others** | 2 |  | 9 | 3 |
| FQ, SXT, Macr, AMG |  |  | 5 | 2 |
| FQ, SXT, Macr, TTC |  |  | 5 | 1 |
| FQ, SXT, TTC, AMG |  |  | 1 | 1 |
| Peni2, Macr, Glyc | 2 |  |  |  |
| CAF, Linco, SXT, Macr |  |  | 2 |  |
| FQ, AMG, Peni, TTC |  |  |  | 2 |
| SXT, Macr, Peni, TTC |  |  | 2 |  |
| Others*** | 2 |  | 7 | 2 |
| FQ, SXT, Macr, AMG, TTC |  |  | 4 | 3 |
| FQ, Linco, SXT, Macr, TTC |  |  | 3 | 1 |
| FQ, Linco, SXT, Macr, TTC |  |  | 3 | 1 |
| CEF, FQ, SXT, Macr, TTC |  |  | 1 | 1 |
| CAF, FQ, Linco, SXT, Macr |  |  | 2 |  |
| CAF, FQ, SXT, Macr, Peni |  |  |  | 2 |
| FQ, SXT, Macr, AMG, Peni |  |  | 1 | 1 |
| Linco, SXT, Macr, Peni, TTC |  |  | 2 |  |
| others**** | 1 |  | 13 | 2 |
| FQ, SXT, Macr, AMG, Peni, TTC |  |  | 4 | 1 |
| FQ, Linco, SXT, Macr, AMG, TTC |  |  | 3 |  |
| others***** |  |  | 5 | 2 |
| CAF, FQ, Linco, SXT, Macr, Peni, TTC |  |  | 2 |  |
| Others****** | 1 |  | 1 | 1 |
| Peni, BLIs, CEF, CEF, CAF, SXT, Macr, AMG, TTC |  |  | 1 |  |

*CoNS: coagulase-negative Staphylococcus

Others:

*FQ, AMG; Linco, Peni; Peni, TTC

**Peni, Macr, TTC; CEF, FQ, SXT; CEF, FQ, TTC; CEF, SXT, Macr; CAF, Linco, SXT; CAF, Macr, TTC; CAF, AMG, Peni; CAF, Macr, AMG; FQ, Linco, SXT; Linco, Macr, AMG; Linco, Macr, TTC; SXT, Macr, Peni; SXT, AMG, TTC; SXT, Peni, TTC

*** Peni, Peni, Linco, SXT; Peni, FQ, Macr, TTC; CEF, CAF, SXT, Macr; CAF, FQ., SXT, Macr; CAF, SXT, Macr, AMG; CAF, FQ, Macr, TTC; FQ., SXT, Macr, Peni; FQ, Linco, Macr, TTC; Linco, SXT, Macr, TTC; SXT, Macr, AMG, TTC

**** Peni, CEF, FQ., SXT, NIT; Peni, CEF, FQ., SXT, NIT; CEF, FQ., SXT, Macr, AMG; CAF, FQ, Linco, Macr, TTC; CAF, Linco, SXT, Macr, Peni; CAF, Linco, Macr, Peni, TTC; CAF, SXT, Macr, AMG, TTC; CAF, SXT, Macr, Peni, TTC; FQ, Linco, SXT, Macr, AMG; FQ, Linco, SXT, Macr, Peni; FQ., SXT, Macr, AMG, Peni; FQ, Linco, SXT, Macr, AMG; FQ, Linco, SXT, Macr, Peni; FQ., SXT, Macr, Peni, TTC; FQ., SXT, Macr, TTC, AMG; FQ., SXT, AMG, Peni, TTC

***** Peni, FQ., SXT, Macr, AMG, TTC; CAF, FQ., SXT, Macr, Peni, TTC; CAF, FQs, Linco, SXT, Macr; CAF, FQ, Linco, SXT, Macr, AMG; CAF, FQ, Linco, SXT, Macr, TTC; CAF, Linco, SXT, Macr, AMG, Peni; FQ., SXT, Macr, AMG, Peni, TTC

******Macr, CAF, FQ, Linco, SXT, Macr, TTC; CEF, CAF, FQ., SXT, AMG2, TTC; CAF, FQ., SXT, Macr, AMG, Peni, TTC

*******Peni, BLIs, CEF2, CAF, SXT, Macr, AMG, TTC
