# Supplementary material for: A Six Years' Trend Analysis of Antimicrobial Resistance Among Bacterial Isolates at Public Health Institute in Amhara Region, Ethiopia
Source: Biomed Res Int. 2025 Jan 29;2025:7676973. doi: 10.1155/bmri/7676973 (PMC11824853; doi:10.1155/bmri/7676973)
Supplement: Supporting Information 2 — Table S2: Distribution of MDR gram-negative bacteria (2016–2021), APHI, Amhara Region, Ethiopia (N = 378). [file 7676973.f2.zip › Supplementary table 2_1.docx]

Supplementary Table 2: Distribution of MDR gram-negative bacteria (2016-2021), at APHI, Northwest Ethiopia (N=378)

| Class of Antibiotics | Type of Bacterial isolates | | | | | | | | | | |
| --- | --- | --- | --- | --- | --- | --- | --- | --- | --- | --- | --- |
|  | E coli (N) | Shigella (N) | Klebsiella (N) | Enterobacter (N) | Citrobacter (N) | Pseudomonas (N) | Proteus (N) | Providencia (N) | Acinetobacter (N) | Serratia (N) | Moraxella (N) |
| FQ., SXT | 7 | 1 |  |  |  |  |  |  |  |  |  |
| SXT, AMG | 1 |  | 5 |  |  |  |  |  |  |  |  |
| Peni, BLIs | 1 |  |  | 1 | 1 |  |  |  |  |  |  |
| CEF, Peni |  |  |  |  | 1 | 3 |  |  |  |  |  |
| BLIs, SXT | 2 | 1 |  |  |  |  |  |  |  |  |  |
| BLIs, TTC | 2 |  |  | 1 |  |  |  |  |  |  |  |
| CEF, SXT |  |  | 1 | 1 |  | 1 |  |  |  |  |  |
| CEF, FQ | 1 | 1 |  |  |  | 1 |  |  |  |  |  |
| SXT, NIT |  |  |  | 1 |  |  |  | 2 |  |  |  |
| SXT, TTC | 3 |  |  |  |  |  |  |  |  |  |  |
| BLIs, CEF | 1 |  |  | 2 | 1 |  |  |  |  |  |  |
| CEF, AMG |  |  | 1 |  |  | 1 |  |  |  |  |  |
| FQ, AMG |  |  |  |  |  | 2 |  |  |  |  |  |
| Others+ | 2 |  | 2 |  |  |  | 1 |  |  |  |  |
| FQ, SXT, AMG | 3 |  | 11 | 3 |  |  |  |  |  |  |  |
| SXT, AMG2 | 1 |  | 4 |  |  |  |  |  |  |  |  |
| CEF, FQ., SXT | 1 |  |  |  |  |  |  |  | 1 |  |  |
| CEF, FQ., AMG |  |  |  |  |  | 3 |  |  |  |  |  |
| CEF, SXT, TTC | 2 |  | 1 |  |  |  |  |  |  |  |  |
| Peni, CEF, SXT | 1 |  | 1 |  |  |  |  |  |  |  |  |
| BLIs, FQ, SXT | 1 |  |  |  | 1 |  |  |  |  |  |  |
| FQ., SXT, TTC | 2 |  |  |  |  |  |  |  |  |  |  |
| FQ, AMG2 |  |  |  |  |  |  |  |  | 1 | 1 |  |
| FQ, Carb, AMG |  |  |  |  |  | 1 |  |  | 1 |  |  |
| SXT, AMG, NIT | 1 |  | 1 |  |  |  |  |  |  |  |  |
| Others++ | 2 |  | 1 | 1 |  |  |  |  | 5 |  |  |
| FQ, SXT, AMG2 |  |  | 10 | 3 |  |  |  |  | 3 | 1 |  |
| Peni, BLIs, SXT, TTC | 3 |  |  |  |  |  |  |  |  |  |  |
| BLIs, CEF, SXT, AMG |  |  | 1 | 1 |  |  |  |  |  |  |  |
| BLIs FQ, SXT, TTC |  |  |  | 1 | 1 |  |  |  |  |  |  |
| CEF2, SXT, TTC |  |  | 1 |  |  |  |  |  | 1 |  |  |
| CEF2, SXT, AMG |  |  | 2 |  |  |  |  |  |  |  |  |
| CEF, FQ, SXT, AMG |  |  | 1 |  |  | 1 |  |  |  |  |  |
| CAF, SXT, AMG2 |  |  | 2 |  |  |  |  |  |  |  |  |
| FQ, SXT, Macr, AMG |  |  | 1 |  |  |  |  |  | 1 |  |  |
| FQ, SXT, AMG, NIT |  |  | 1 |  |  |  |  |  |  | 1 |  |
| Others+++ | 8 |  | 8 |  | 4 | 1 |  |  | 3 | 1 |  |
| FQ, SXT, AMG2, Carb |  |  | 1 | 1 |  |  |  |  | 3 |  |  |
| BLIs, CEF, FQ, SXT, AMG | 1 |  | 2 | 1 |  |  |  |  |  |  |  |
| BLIs CEF, SXT, AMG, TTC |  |  | 1 | 3 |  |  |  |  |  |  |  |
| CEF2, FQ, SXT, AMG | 1 |  |  | 1 |  |  |  |  | 1 |  |  |
| CEF, FQ, SXT, AMG2 |  |  | 3 |  |  |  |  |  |  |  |  |
| Peni, BLIs CEF, FQ, TTC |  |  | 1 |  | 1 |  |  |  |  |  |  |
| CEF, FQ, AMG2, Carb |  |  |  |  |  | 2 |  |  |  |  |  |
| BLIs, CEF, FQ, SXT, TTC | 1 |  | 1 |  |  |  |  |  |  |  |  |
| BLIs, CEF, SXT, AMG 2 |  |  | 2 |  |  |  |  |  |  |  |  |
| CEF, FQ, SXT, AMG, TTC |  |  | 1 |  |  |  |  |  | 1 |  |  |
| FQ, Linco, SXT, Macr, Peni |  |  | 2 |  |  |  |  |  |  |  |  |
| Others++++ |  |  | 1 |  |  |  |  |  | 1 |  |  |
| BLIs, CEF, FQ., SXT, AMG, TTC |  |  | 5 |  |  |  |  |  |  |  |  |
| BLIs, CEF, SXT, AMG2, TTC |  |  | 2 | 2 |  |  |  |  |  |  |  |
| CEF, SXT, AMG2, TTC | 2 |  | 2 |  |  |  |  |  |  |  |  |
| Peni, BLIs, CEF, FQ., SXT, AMG | 1 |  | 2 |  |  |  |  |  |  |  |  |
| Peni, BLIs, CEF, SXT, AMG2 |  |  | 1 | 1 |  |  |  |  |  |  |  |
| BLIs, CEF, FQ., SXT, AMG2 | 1 |  | 2 |  |  |  |  |  |  |  |  |
| BLIs, CEF, FQ., SXT, TTC, AMG | 1 |  |  | 1 |  |  |  |  | 1 |  |  |
| CEF2, FQ., SXT, AMG2 | 1 |  | 2 |  |  |  |  |  |  |  |  |
| BLIs, CEF2, FQ., SXT, TTC | 3 |  |  |  |  |  |  |  |  |  |  |
| BLIs, CEF2, SXT, TTC, AMG |  |  | 2 |  |  |  |  |  | 1 |  |  |
| BLIs, FQ, SXT, AMG2, TTC | 1 |  |  | 1 |  |  |  |  |  |  | 1 |
| Others+++++ | 5 |  | 5 |  |  |  | 1 |  | 5 |  |  |
| Peni, BLIs, CEF, FQ, SXT, AMG2 |  |  | 12 |  | 1 |  |  |  |  |  |  |
| BLIs, CEF2, FQ, SXT, TTC, AMG | 4 |  | 1 |  |  |  |  |  |  |  |  |
| BLIs, CEF2, SXT, AMG2, TTC |  |  | 5 |  |  |  |  |  |  |  |  |
| BLIs, CEF2, FQ, SXT, AMG, TTC | 1 |  | 1 |  |  |  |  |  | 1 |  |  |
| BLIs, CEF, FQ, SXT, AMG2, TTC |  |  | 2 |  | 1 |  |  |  |  |  |  |
| CEF2, FQ, SXT, AMG2, TTC |  |  |  |  |  |  |  |  | 2 |  |  |
| Others++++++ | 3 |  | 5 | 8 | 2 |  | 1 |  | 3 | 1 |  |
| Peni, BLIs, CEF, FQ, SXT, AMG2, TTC | 2 |  | 4 | 2 |  |  |  |  |  |  |  |
| BLIs, CEF2, FQ, SXT, AMG2, TTC |  |  | 4 | 2 |  |  |  |  |  |  |  |
| Peni, BLIs, CEF2, FQ, SXT, AMG2 |  |  | 4 |  |  |  |  |  |  |  |  |
| Peni, BLIs, CEF, CEF, FQ, SXT, TTC, AMG | 1 |  | 1 |  |  |  |  |  |  |  |  |
| Peni, BLIs, CEF, CAF, SXT, AMG2, TTC |  |  | 1 | 1 |  |  |  |  |  |  |  |
| Peni, BLIs, CEF, FQ., SXT, AMG2, |  |  | 1 | 1 |  |  |  |  |  |  |  |
| Others+++++++ | 4 |  | 6 | 3 | 1 |  |  |  | 2 |  |  |
| Peni, BLIs, CEF2, FQ, SXT, AMG2, TTC | 2 |  | 1 |  |  |  |  |  |  |  |  |
| Peni, BLIs, CEF, FQ, SXT, AMG2, Carb, TTC |  |  | 2 |  |  |  |  |  |  |  |  |
| Others++++++++ | 2 |  | 2 |  |  |  |  |  | 2 |  |  |
| Peni, BLIs, CEF3, FQ, SXT, AMG2, TTC; Peni, BLIs, CEF3, FQ2, SXT, AMG2, |  |  | 1 | 1 |  |  |  |  |  |  |  |

*** Others+: BLIs, FQ; CEF2; CEF, TTC; FQ, NIT; AMG2

- **Others++:** Peni, BLIs, SXT; BLIs, FQ, NIT; BLIs, SXT, NIT; BLIs, TTC, AMG; BLIs, CEF, CAF; CEF2, NIT; CEF2, TTC; CEF, AMG2; FQ, SXT, NIT;
- **Others+++:** Peni, BLIs, CEF, SXT; Peni, BLIs, CEF, TTC; Peni, BLIs, SXT, AMG; Peni, CEF, SXT, AMG; Peni, CEF, FQ , SXT; Peni, CE, AMG, TTC; Peni, FQ, SXT, NIT; Peni, FQ, SXT, TTC; BLIs, CEF2, SXT; BLIs, CEF2, AMG; BLIs, CEF, SXT, NIT; BLIs, CEF, SXT, TTC; BLIs, CEF, FQ, SXT; BLIs, FQ, AMG2; BLIs, SXT, TTC, AMG; CEF, FQ, SXT, NIT; CEF, SXT, AMG, TTC; CEF, SXT, AMG2; CEF, SXT, Macr, AMG; CAF, FQ, SXT, Macr; CAF, FQ, SXT, TTC; FQ, AMG2, NIT; FQ, SXT, TTC, AMG; FQ, AMG, Carb, AMG; Peni, BLIs, CEF2, FQ
- **Others++++:** Peni, BLIs, FQ, SXT, AMG; Peni, BLIs CEF, AMG, TTC; Peni, CEF, CAF, FQ, SXT; BLIs, CEF2, SXT, AMG; Peni, CEF, FQ, SXT, AMG; Peni, CAF, SXT, AMG, TTC; Peni, FQ, SXT, AMG, TTC; BLIs, CEF2, FQ, SXT; BLIs, CEF2, SXT, AMG; BLIs, CEF2, NIT, TTC; BLIs, CEF, CAF, SXT, TTC; BLIs, CEF, FQ, AMG, TTC; BLIs, CEF, SXT, AMG, TTC; BLIs, FQ, SXT, TTC, AMG; BLIs, SXT, AMG, NIT, FQ; CEF2, FQ, SXT, TTC; CEF2, FQ, AMG , TTC; CEF2, SXT, AMG, TTC; CEF2, SXT, AMG 2; CEF, FQ, SXT, TTC, AMG; CEF, FQ, SXT, NIT, TTC; CEF, FQ, AMG2, TTC; CAF, FQ, SXT, TTC, AMG; FQ, AMG2, Carb, NIT
- **Others+++++:** Peni, BLIs, CEF, FQ., SXT, TTC; Peni, BLIs, CEF, FQ., TTC, AMG; Peni, BLIs, CEF, SXT, AMG, TTC; Peni, CEF, F.Q., SXT, AMG, TTC; BLIs CEF2, SXT, AMG, TTC; Peni, CEF, FQ., SXT, AMG2; Peni, BLIs, SXT, AMG2, Carb; Peni, BLIs, FQ, SXT, NIT, TTC; BLIs, CEF, CAF, FQ., SXT, AMG; BLIs, CEF2, FQ., SXT, AMG; BLIs, CEF2, FQ., SXT, AMG; BLIs, CEF2, SXT, AMG, TTC; BLIs, CEF2, SXT, AMG2; BLIs, CEF, FQ., SXT, AMG, Carb; CEF2, CAF, FQ., SXT, TTC; CEF2, FQ., SXT, AMG, TTC; CEF2, SXT, AMG2, TTC; BLIs, CEF, FQ., SXT, AMG, Carb
- **Others++++++:** Peni, BLIs CEF, FQ., SXT, AMG, Carb; Peni, BLIs, CEF2, FQ., TTC, AMG; Peni, BLIs, CEF, FQ., SXT, TTC, AMG; Peni, BLIs, CEF, SXT, AMG2, TTC; Peni, CEF2, CAF, SXT, AMG2; Peni, CEF2, FQ., SXT, AMG2;Peni, CEF, CAF, FQ., SXT, AMG2; BLIs, CEF3, FQ., SXT, AMG ; BLIs, CEF2, FQ., SXT, AMG2; BLIs, CEF2, FQ., SXT, Carb, AMG; BLIs CEF2, FQ., SXT, NIT, TTC; BLIs, CEF2, FQ., SXT, Carb, AMG; BLIs, CEF, FQ., SXT, AMG, Carb, Peni;BLIs, CEF, FQ., SXT, AMG, NIT, AMG;BLIs, CEF, FQ., SXT, NIT, TTC, AMG; CEF2, CAF, FQ., SXT, AMG 2; CEF2, FQ., SXT, AMG, Carb, TTC ; CEF, CAF, FQ., SXT, AMG, Carb, TTC; CEF, SXT, AMG2, Carb, NIT, TTC; CEF, FQ., SXT, AMG, Carb, TTC;CEF, FQ., SXT, AMG, BLIs, TTC; CEF, FQ., SXT, TTC, AMG2; CEF, FQ., SXT, AMG2, Carb; Peni, BLIs CEF, CEF2, NIT, TTC
- **Others+++++++:** Peni, BLIs, CEF2, FQ., SXT, TTC, AMG; Peni, BLIs, CEF2, SXT, AMG, TTC, AMG; Peni, BLIs, CEF, CAF, FQ., SXT, AMG, Carb; Peni, BLIs, CEF, CAF, FQ., SXT, AMG2; Peni, BLIs, CEF, FQ., SXT, AMG, NIT, TTC; Peni, BLIs, CEF, FQ., SXT, AMG2, NIT; Peni, BLIs, CEF, FQ., SXT, TTC, AMG; Peni, BLIs CAF, FQ., SXT, AMG2, TTC; Peni, CEF2, CAF, FQ., SXT, AMG, TTC; BLIs CEF2, FQ., SXT, AMG, Carb, TTC; BLIs, CEF2, FQ., AMG2, NIT, TTC; BLIs, CEF, FQ., SXT, AMG2, Carb, TTC; BLIs, CEF, FQ., SXT, AMG2, NIT, TTC; CEF2, CAF, FQ., SXT, AMG, Carb, TTC; CEF2, FQ., SXT, AMG2, Carb, BLIs; CEF2, FQ., SXT, AMG2, BLIs, TTC
- Others++++++++: Peni, BLIs, CEF2, CAF, FQ, SXT, AMG2; Peni, BLIs, CEF2, FQ, SXT, AMG2, NIT; Peni, BLIs, CEF2, FQ, SXT, NIT, TTC, AMG; BLIs, CEF2, FQ, SXT, AMG2, Carb, TTC; BLIs, CEF2, FQ, SXT, AMG2, NIT, TTC; CEF2, FQ, SXT, AMG2, Carb, NIT, TTC
- The numbers associated with antibiotic categories indicate the number of antibiotics in that category involved in drug resistance.
