# Supplementary material for: A Six Years' Trend Analysis of Antimicrobial Resistance Among Bacterial Isolates at Public Health Institute in Amhara Region, Ethiopia
Source: Biomed Res Int. 2025 Jan 29;2025:7676973. doi: 10.1155/bmri/7676973 (PMC11824853; doi:10.1155/bmri/7676973)
Supplement: Supporting Information 3 — Table S3: Distribution of MDR gram-negative bacteria (2016–2021), APHI, Amhara Region. [file 7676973.f3.docx]

Supplementary Table 3: Distribution of MDR gram-negative bacteria (2016-2021), at APHI, Northwest Ethiopia

| Antibiotics | Type of bacteria | | | | | | | | | | | | | | | |
| --- | --- | --- | --- | --- | --- | --- | --- | --- | --- | --- | --- | --- | --- | --- | --- | --- |
|  | A baumannii | Enterobacter cloacae | E coli | K pneumoniae | Citrobacter species | Enterobacter aerogenes | Klebsiella species | Moraxella catarrhalis | Morganella morganii | Proteus species | Providencia species | Pseudomonas aeruginosa | Pseudomonas alcaligenes | Salmonella choleraesuis | Serratia marcescens | Shigella dysenteriae |
| Ampicillin, Augmentin |  | 2 | 1 |  | 1 |  |  |  |  |  |  |  |  |  |  |  |
| Augmentin, Cefoxitin |  | 2 |  |  |  |  |  |  |  |  |  |  |  |  |  |  |
| Augmentin, Ciprofloxacin |  |  | 1 |  |  |  |  |  |  |  |  |  |  |  |  |  |
| Augmentin, Cotrimoxazole |  |  | 2 | 1 |  |  |  |  |  |  |  |  |  |  |  |  |
| Augmentin, Tetracycline |  |  | 2 |  |  | 1 |  |  |  |  |  |  |  |  |  |  |
| Ceftazidime, Ceftriaxone |  |  |  |  |  |  |  |  |  | 1 |  |  |  |  |  |  |
| Ceftazidime, Cotrimoxazole |  |  |  |  |  |  |  |  |  |  |  | 1 |  |  |  |  |
| Ceftazidime, Piperacillin | 1 |  |  |  |  |  |  |  |  |  |  | 2 |  |  |  |  |
| Ceftazidime, Piperacillin |  |  |  |  |  |  |  |  |  |  |  | 1 |  |  |  |  |
| Ceftazidime, Tobramycin |  |  |  |  |  |  |  |  |  |  |  | 1 |  |  |  |  |
| Ceftriaxone, Ciprofloxacin | 1 |  | 1 |  |  |  |  |  |  |  |  |  |  |  |  |  |
| Ceftriaxone, Cotrimoxazole |  |  |  | 1 |  | 1 |  |  |  |  |  |  |  |  |  |  |
| Ceftriaxone, Gentamicin |  |  |  | 1 |  |  |  |  |  |  |  |  |  |  |  |  |
| Ceftriaxone, Tetracycline |  |  |  |  |  |  | 1 |  |  |  |  |  |  |  |  |  |
| Cefuroxime, Ciprofloxacin |  |  |  |  |  |  |  |  |  |  |  |  |  | 1 |  |  |
| Chloramphenicol, Erythromycin |  |  |  |  |  |  |  |  |  |  |  |  |  |  |  |  |
| Ciprofloxacin, Cotrimoxazole |  |  | 7 |  | 0 | 0 | 0 | 0 | 0 | 0 | 0 | 0 | 0 | 0 | 0 | 1 |
| Ciprofloxacin, Nitrofurantoin |  |  | 1 |  |  |  |  |  |  |  |  |  |  |  |  |  |
| Ciprofloxacin, Gentamicin |  |  |  |  |  |  |  |  |  |  |  | 2 |  |  |  |  |
| Cotrimoxazole, Erythromycin |  |  |  |  |  |  |  | 1 |  |  |  |  |  |  |  |  |
| Cotrimoxazole, Gentamicin |  |  | 1 | 4 |  |  | 1 |  |  |  |  |  |  |  |  |  |
| Cotrimoxazole, Nitrofurantoin |  | 1 |  |  |  |  |  |  |  |  | 2 |  |  |  |  |  |
| Cotrimoxazole, Tetracycline |  |  | 3 |  |  |  |  |  |  |  |  |  |  |  |  |  |
| Gentamicin, Tobramycin |  |  |  | 1 |  |  |  |  |  |  |  |  |  |  |  |  |
| Ampicillin, Augmentin, Cotrimoxazole |  |  | 1 |  |  |  |  |  |  |  |  |  |  |  |  |  |
| Ampicillin, Ceftriaxone, Cotrimoxazole |  |  | 1 |  |  |  |  |  |  |  |  |  |  |  |  |  |
| Ampicillin, Ceftriaxone, Cotrimoxazole |  |  |  |  |  |  | 1 |  |  |  |  |  |  |  |  |  |
| Augmentin, Ciprofloxacin, Cotrimoxazole |  |  | 1 |  |  |  |  |  |  |  |  |  |  |  |  |  |
| Augmentin, Ciprofloxacin, Nitrofurantoin |  | 1 |  |  |  |  |  |  |  |  |  |  |  |  |  |  |
| Augmentin, Cotrimoxazole, Nitrofurantoin | 1 |  |  |  |  |  |  |  |  |  |  |  |  |  |  |  |
| Augmentin, Tetracycline, Tobramycin |  |  |  | 1 |  |  |  |  |  |  |  |  |  |  |  |  |
| Augmentin, Ceftriaxone, Chloramphenicol |  |  |  |  |  |  |  |  |  | 1 |  |  |  |  |  |  |
| Augmentin, Ciprofloxacin, Cotrimoxazole |  |  |  |  | 1 |  |  |  |  |  |  |  |  |  |  |  |
| Ceftazidime, Ceftriaxone, Nitrofurantoin | 1 |  |  |  |  |  |  |  |  |  |  |  |  |  |  |  |
| Ceftazidime, Ceftriaxone, Tetracycline | 1 |  |  |  |  |  |  |  |  |  |  |  |  |  |  |  |
| Ceftazidime, Gentamicin, Tobramycin | 1 |  |  |  |  |  |  |  |  |  |  |  |  |  |  |  |
| Ceftriaxone, Ciprofloxacin, Cotrimoxazole | 2 |  |  |  |  |  |  |  |  |  |  |  |  |  |  |  |
| Ceftazidime, Ciprofloxacin, Gentamicin |  |  |  |  |  |  |  |  |  |  |  | 2 |  |  |  |  |
| Ceftazidime, Ciprofloxacin, Tobramycin |  |  |  |  |  |  |  |  |  |  |  | 1 |  |  |  |  |
| Ceftriaxone, Cotrimoxazole, Tetracycline |  |  | 2 | 1 |  |  |  |  |  |  |  |  |  |  |  |  |
| Ceftriaxone, Norfloxacin, Sulfamethoxazole |  |  | 1 |  |  |  |  |  |  |  |  |  |  |  |  |  |
| Ciprofloxacin, Cotrimoxazole, Gentamicin |  | 3 | 2 | 10 |  |  |  |  |  |  |  |  |  |  |  |  |
| Ciprofloxacin, Cotrimoxazole, Nitrofurantoin |  |  | 1 |  |  |  |  |  |  |  |  |  |  |  |  |  |
| Ciprofloxacin, Cotrimoxazole, Tetracycline |  |  | 2 |  |  |  |  |  |  |  |  |  |  |  |  |  |
| Ciprofloxacin, Cotrimoxazole, Tobramycin |  |  | 1 | 1 |  |  |  |  |  |  |  |  |  |  |  |  |
| Ciprofloxacin, Gentamicin, Tobramycin | 1 |  |  |  |  |  |  |  |  |  |  |  |  |  | 1 |  |
| Ciprofloxacin, Imipenem, Tobramycin | 1 |  |  |  |  |  |  |  |  |  |  |  |  |  |  |  |
| Ciprofloxacin, Clindamycin, Cotrimoxazole |  |  |  |  |  |  |  |  |  |  |  |  |  |  |  |  |
| Ciprofloxacin, Gentamicin, Imipenem |  |  |  |  |  |  |  |  |  |  |  | 1 |  |  |  |  |
| Cotrimoxazole, Gentamicin, Nitrofurantoin |  |  | 1 |  |  |  |  |  |  |  |  |  |  |  |  |  |
| Cotrimoxazole, Gentamicin, Tobramycin |  |  | 1 | 4 |  |  |  |  |  |  |  |  |  |  |  |  |
| Cotrimoxazole, Nitrofurantoin, Tobramycin |  |  |  | 1 |  |  |  |  |  |  |  |  |  |  |  |  |
| Ampicillin, Augmentin, Ceftriaxone, Cotrimoxazole |  |  |  | 1 |  |  |  |  |  |  |  |  |  |  |  |  |
| Ampicillin, Augmentin, Ceftriaxone, Tetracycline |  |  | 1 |  |  |  |  |  |  |  |  |  |  |  |  |  |
| Ampicillin, Augmentin, Cotrimoxazole, Tetracycline |  |  | 2 |  |  |  |  |  |  |  |  |  |  |  |  |  |
| Ampicillin, Augmentin, Cotrimoxazole, Tobramycin |  |  | 1 |  |  |  |  |  |  |  |  |  |  |  |  |  |
| Ampicillin, Ceftriaxone, Cotrimoxazole, Gentamicin |  |  |  | 1 |  |  |  |  |  |  |  |  |  |  |  |  |
| Ampicillin, Augmentin, Cotrimoxazole, Tetracycline |  |  |  |  | 1 |  |  |  |  |  |  |  |  |  |  |  |
| Ampicillin, Ceftriaxone, Ciprofloxacin, Cotrimoxazole |  |  |  |  |  |  |  |  |  |  |  |  |  |  | 1 |  |
| Ampicillin, Ceftriaxone, Gentamicin, Tetracycline |  |  |  |  | 1 |  |  |  |  |  |  |  |  |  |  |  |
| Ampicillin, Ciprofloxacin, Cotrimoxazole, Nitrofurantoin |  |  | 1 |  |  |  |  |  |  |  |  |  |  |  |  |  |
| Ampicillin, Ciprofloxacin, Cotrimoxazole, Tetracycline |  |  | 1 |  |  |  |  |  |  |  |  |  |  |  |  |  |
| Augmentin, Ceftazidime, Ceftriaxone, Cotrimoxazole |  |  |  | 1 |  |  |  |  |  |  |  |  |  |  |  |  |
| Augmentin, Ceftazidime, Ceftriaxone, Gentamicin |  |  | 1 |  |  |  |  |  |  |  |  |  |  |  |  |  |
| Augmentin, Ceftazidime, Cotrimoxazole, Nitrofurantoin |  |  |  |  | 1 |  |  |  |  |  |  |  |  |  |  |  |
| Augmentin, Ceftazidime, Cotrimoxazole, Tetracycline |  |  |  |  | 1 |  |  |  |  |  |  |  |  |  |  |  |
| Augmentin, Ceftriaxone, Ciprofloxacin, Cotrimoxazole |  |  | 1 |  |  |  |  |  |  |  |  |  |  |  |  |  |
| Augmentin, Ceftriaxone, Cotrimoxazole, Gentamicin |  |  |  | 1 |  | 1 |  |  |  |  |  |  |  |  |  |  |
| Augmentin, Ciprofloxacin, Gentamicin, Tobramycin |  |  | 1 |  |  |  |  |  |  |  |  |  |  |  |  |  |
| Augmentin, Cotrimoxazole, Tetracycline, Tobramycin |  |  | 1 |  |  |  |  |  |  |  |  |  |  |  |  |  |
| Augmentin, Ciprofloxacin, Cotrimoxazole, Tetracycline |  |  |  |  | 1 | 1 |  |  |  |  |  |  |  |  |  |  |
| Ceftazidime, Ceftriaxone, Cotrimoxazole, Tetracycline | 1 |  |  | 1 |  |  |  |  |  |  |  |  |  |  |  |  |
| Ceftazidime, Ceftriaxone, Cotrimoxazole, Gentamicin |  |  |  |  |  |  | 1 |  |  |  |  |  |  |  |  |  |
| Ceftazidime, Ceftriaxone, Cotrimoxazole, Tobramycin |  |  |  |  |  |  | 1 |  |  |  |  |  |  |  |  |  |
| Ceftazidime, Ciprofloxacin, Cotrimoxazole, Gentamicin |  |  |  |  |  |  | 1 |  |  |  |  | 1 |  |  |  |  |
| Ceftriaxone, Ciprofloxacin, Cotrimoxazole, Nitrofurantoin |  |  |  | 1 |  |  |  |  |  |  |  |  |  |  |  |  |
| Ceftriaxone, Cotrimoxazole, Gentamicin, Tetracycline |  |  |  | 1 |  |  |  |  |  |  |  |  |  |  |  |  |
| Ceftriaxone, Cotrimoxazole, Gentamicin, Tobramycin |  |  |  | 1 |  |  |  |  |  |  |  |  |  |  |  |  |
| Ceftriaxone, Cotrimoxazole, Erythromycin, Gentamicin |  |  |  |  |  | 1 |  |  |  |  |  |  |  |  |  |  |
| Chloramphenicol, Ciprofloxacin, Cotrimoxazole, Erythromycin |  | 1 |  |  |  |  |  |  |  |  |  |  |  |  |  |  |
| Chloramphenicol, Cotrimoxazole, Gentamicin, Tobramycin |  |  |  | 2 |  |  |  |  |  |  |  |  |  |  |  |  |
| Chloramphenicol, Ciprofloxacin, Cotrimoxazole, Tetracycline |  |  |  |  |  |  |  |  |  | 1 |  |  |  |  |  |  |
| Ciprofloxacin, Cotrimoxazole, Gentamicin, Imipenem | 1 | 1 |  |  |  |  |  |  |  |  |  |  |  |  |  |  |
| Ciprofloxacin, Cotrimoxazole, Gentamicin, Nitrofurantoin |  |  |  |  |  |  |  |  |  |  |  |  |  |  | 1 |  |
| Ciprofloxacin, Gentamicin, Nitrofurantoin, Tobramycin |  |  |  |  |  |  |  |  |  |  |  | 1 |  |  |  |  |
| Ciprofloxacin, Cotrimoxazole, Gentamicin, Tobramycin | 3 | 3 | 2 | 10 |  |  |  |  |  |  |  |  |  |  | 1 |  |
| Ciprofloxacin, Cotrimoxazole, Tetracycline, Tobramycin |  |  | 1 |  |  |  |  |  |  |  |  |  |  |  |  |  |
| Ciprofloxacin, Gentamicin, Imipenem, Tobramycin |  |  |  | 1 |  |  |  |  |  |  |  |  |  |  |  |  |
| Ciprofloxacin, Gentamicin, Nitrofurantoin, Sulfamethoxazole |  |  | 1 |  |  |  |  |  |  |  |  |  |  |  |  |  |
| Ampicillin, Augmentin, Ceftazidime, Ceftriaxone, Ciprofloxacin |  |  | 1 |  |  |  |  |  |  |  |  |  |  |  |  |  |
| Ampicillin, Augmentin, Ceftriaxone, Ciprofloxacin, Tetracycline |  |  |  |  | 1 |  | 1 |  |  |  |  |  |  |  |  |  |
| Ampicillin, Augmentin, Ciprofloxacin, Cotrimoxazole, Gentamicin |  |  | 1 |  |  |  |  |  |  |  |  |  |  |  |  |  |
| Ampicillin, Augmentin, Ceftriaxone, Gentamicin, Tetracycline |  |  |  |  |  | 1 |  |  |  |  |  |  |  |  |  |  |
| Ampicillin, Ceftazidime, Chloramphenicol, Ciprofloxacin, Cotrimoxazole |  |  | 1 |  |  |  |  |  |  |  |  |  |  |  |  |  |
| Augmentin, Ceftazidime, Ceftriaxone, Cotrimoxazole, Gentamicin |  |  |  |  |  |  |  |  |  | 1 |  |  |  |  |  |  |
| Ampicillin, Ceftriaxone, Ciprofloxacin, Cotrimoxazole, Gentamicin |  |  |  | 1 |  |  |  |  |  |  |  |  |  |  |  |  |
| Ampicillin, Chloramphenicol, Cotrimoxazole, Gentamicin, Tetracycline |  |  |  | 1 |  |  |  |  |  |  |  |  |  |  |  |  |
| Ampicillin, Ciprofloxacin, Cotrimoxazole, Gentamicin, Tetracycline |  |  |  | 1 |  |  |  |  |  |  |  |  |  |  |  |  |
| Augmentin, Ceftazidime, Ceftriaxone, Ciprofloxacin, Cotrimoxazole |  |  |  | 1 |  |  |  |  |  |  |  |  |  |  |  |  |
| Augmentin, Ceftazidime, Ceftriaxone, Cotrimoxazole, Gentamicin |  |  |  | 1 |  |  |  |  |  |  |  |  |  |  |  |  |
| Augmentin, Ceftazidime, Ceftriaxone, Nitrofurantoin, Tetracycline |  |  |  |  | 1 |  |  |  |  |  |  |  |  |  |  |  |
| Ceftazidime, Ciprofloxacin, Gentamicin, Imipenem, Tobramycin |  |  |  |  |  |  |  |  |  |  |  | 2 |  |  |  |  |
| Augmentin, Ceftriaxone, Ciprofloxacin, Cotrimoxazole, Gentamicin |  |  |  | 1 |  | 1 |  |  |  |  |  |  |  |  |  |  |
| Augmentin, Ceftriaxone, Cotrimoxazole, Gentamicin, Tetracycline |  |  |  |  |  | 3 |  |  |  |  |  |  |  |  |  |  |
| Augmentin, Ceftriaxone, Chloramphenicol, Cotrimoxazole, Tetracycline |  |  |  |  |  |  |  |  |  |  | 1 |  |  |  |  |  |
| Augmentin, Ceftriaxone, Ciprofloxacin, Cotrimoxazole, Tetracycline |  |  | 1 |  |  |  | 1 |  |  |  |  |  |  |  |  |  |
| Augmentin, Ceftriaxone, Ciprofloxacin, Cotrimoxazole, Tobramycin |  | 1 | 1 |  |  |  |  |  |  |  |  |  |  |  |  |  |
| Augmentin, Ceftriaxone, Ciprofloxacin, Gentamicin, Tetracycline |  |  |  | 1 |  |  |  |  |  |  |  |  |  |  |  |  |
| Augmentin, Ceftriaxone, Cotrimoxazole, Gentamicin, Tetracycline |  |  |  | 1 |  |  |  |  |  |  |  |  |  |  |  |  |
| Augmentin, Ceftriaxone, Cotrimoxazole, Gentamicin, Tobramycin |  |  |  | 2 |  |  |  |  |  |  |  |  |  |  |  |  |
| Augmentin, Ciprofloxacin, Cotrimoxazole, Tetracycline, Tobramycin |  |  | 1 |  |  |  |  |  |  |  |  |  |  |  |  |  |
| Augmentin, Cotrimoxazole, Gentamicin, Nitrofurantoin, Norfloxacin |  |  | 1 |  |  |  |  |  |  |  |  |  |  |  |  |  |
| Ceftazidime, Ceftriaxone, Ciprofloxacin, Cotrimoxazole, Gentamicin |  | 1 | 1 |  |  |  |  |  |  |  |  |  |  |  |  |  |
| Ceftazidime, Ceftriaxone, Ciprofloxacin, Cotrimoxazole, Tobramycin | 1 |  |  |  |  |  |  |  |  |  |  |  |  |  |  |  |
| Ceftazidime, Ceftriaxone, Ciprofloxacin, Cotrimoxazole, Tetracycline | 1 |  |  |  |  |  |  |  |  |  |  |  |  |  |  |  |
| Ceftazidime, Ceftriaxone, Ciprofloxacin, Gentamicin, Tetracycline |  |  |  | 1 |  |  |  |  |  |  |  |  |  |  |  |  |
| Ceftazidime, Ceftriaxone, Cotrimoxazole, Gentamicin, Tetracycline | 1 |  |  |  |  |  |  |  |  |  |  |  |  |  |  |  |
| Ceftazidime, Ceftriaxone, Cotrimoxazole, Gentamicin, Tobramycin | 1 |  |  |  |  |  |  |  |  |  |  |  |  |  |  |  |
| Ceftazidime, Ciprofloxacin, Cotrimoxazole, Gentamicin, Tetracycline |  |  |  | 1 |  |  |  |  |  |  |  |  |  |  |  |  |
| Ceftriaxone, Ciprofloxacin, Cotrimoxazole, Gentamicin, Tetracycline |  |  |  | 1 |  |  |  |  |  |  |  |  |  |  |  |  |
| Ceftriaxone, Ciprofloxacin, Cotrimoxazole, Gentamicin, Tobramycin |  |  |  | 3 |  |  |  |  |  |  |  |  |  |  |  |  |
| Ceftriaxone, Ciprofloxacin, Cotrimoxazole, Tetracycline, Tobramycin | 1 |  |  |  |  |  |  |  |  |  |  |  |  |  |  |  |
| Ceftriaxone, Ciprofloxacin, Cotrimoxazole, Nitrofurantoin, Tetracycline |  |  |  |  |  |  |  |  |  |  |  |  | 1 |  |  |  |
| Ceftriaxone, Cotrimoxazole, Gentamicin, Tetracycline, Tobramycin | 1 |  |  | 1 |  |  |  |  |  |  |  |  |  |  |  |  |
| Ceftriaxone, Ciprofloxacin, Gentamicin, Tetracycline, Tobramycin | 1 |  |  |  |  |  |  |  |  |  |  |  |  |  |  |  |
| Chloramphenicol, Ciprofloxacin, Cotrimoxazole, Tetracycline, Tobramycin |  |  |  |  | 1 |  |  |  |  |  |  |  |  |  |  |  |
| Ciprofloxacin, Clindamycin, Cotrimoxazole, Erythromycin, Oxacillin |  |  |  | 2 |  |  |  |  |  |  |  |  |  |  |  |  |
| Ciprofloxacin, Cotrimoxazole, Gentamicin, Imipenem, Tobramycin | 1 | 1 |  |  |  |  |  |  |  |  |  |  |  |  |  |  |
| Ciprofloxacin, Cotrimoxazole, Gentamicin, Meropenem, Tobramycin | 1 |  |  |  |  |  |  |  |  |  |  |  |  |  |  |  |
| Ciprofloxacin, Cotrimoxazole, Gentamicin, Imipenem, Tobramycin |  |  |  |  |  |  | 1 |  |  |  |  |  |  |  |  |  |
| Ciprofloxacin, Gentamicin, Imipenem, Nitrofurantoin, Tobramycin |  |  |  |  | 1 |  |  |  |  |  |  |  |  |  |  |  |
| Ampicillin, Augmentin, Ceftriaxone, Ciprofloxacin, Cotrimoxazole, Tetracycline |  |  | 1 |  |  |  |  |  |  |  |  |  |  |  |  |  |
| Ampicillin, Augmentin, Ceftriaxone, Ciprofloxacin, Cotrimoxazole, Tobramycin |  |  | 2 | 1 |  |  |  |  |  |  |  |  |  |  |  |  |
| Ampicillin, Augmentin, Ceftriaxone, Ciprofloxacin, Tetracycline, Tobramycin |  |  | 1 |  |  |  |  |  |  |  |  |  |  |  |  |  |
| Ampicillin, Augmentin, Ceftriaxone, Cotrimoxazole, Gentamicin, Tetracycline |  |  | 1 |  |  |  |  |  |  |  |  |  |  |  |  |  |
| Ampicillin, Augmentin, Ceftriaxone, Cotrimoxazole, Gentamicin, Tobramycin |  | 1 |  | 1 |  |  | 1 |  |  |  |  |  |  |  |  |  |
| Ampicillin, Ceftriaxone, Ciprofloxacin, Cotrimoxazole, Gentamicin, Tetracycline |  |  |  |  |  |  | 1 |  | 1 |  |  |  |  |  |  |  |
| Augmentin, Ceftazidime, Ceftriaxone, Cotrimoxazole, Gentamicin, Tetracycline |  |  |  |  |  |  | 1 |  |  |  |  |  |  |  |  |  |
| Ampicillin, Ceftriaxone, Ciprofloxacin, Cotrimoxazole, Gentamicin, Tobramycin |  |  | 1 |  |  |  |  |  |  |  |  |  |  |  |  |  |
| Ampicillin, Augmentin, Cotrimoxazole, Gentamicin, Meropenem, Tobramycin |  |  |  |  | 1 |  |  |  |  |  |  |  |  |  |  |  |
| Ampicillin, Augmentin, Ciprofloxacin, Cotrimoxazole, Nitrofurantoin, Tetracycline |  |  |  |  |  |  |  |  |  | 1 |  |  |  |  |  |  |
| Augmentin, Cefoxitin, Chloramphenicol, Ciprofloxacin, Cotrimoxazole, Gentamicin |  |  | 1 |  |  |  |  |  |  |  |  |  |  |  |  |  |
| Augmentin, Ceftazidime, Ceftriaxone, Ciprofloxacin, Cotrimoxazole, Gentamicin |  |  |  |  |  |  | 1 |  |  |  |  |  |  |  |  |  |
| Augmentin, Ceftazidime, Ceftriaxone, Ciprofloxacin, Cotrimoxazole, Tetracycline |  |  | 2 |  |  |  |  |  |  |  |  |  |  |  |  |  |
| Augmentin, Ceftazidime, Ceftriaxone, Ciprofloxacin, Cotrimoxazole, Tobramycin |  |  | 1 |  |  |  |  |  |  |  |  |  |  |  |  |  |
| Augmentin, Ceftazidime, Ceftriaxone, Cotrimoxazole, Gentamicin, Tetracycline | 1 |  |  |  |  |  |  |  |  |  |  |  |  |  |  |  |
| Augmentin, Ceftazidime, Ceftriaxone, Cotrimoxazole, Gentamicin, Tobramycin |  |  |  | 1 |  |  |  |  |  |  |  |  |  |  |  |  |
| Augmentin, Ceftazidime, Ceftriaxone, Cotrimoxazole, Tetracycline, Tobramycin |  |  |  | 1 |  |  |  |  |  |  |  |  |  |  |  |  |
| Augmentin, Ceftazidime, Ciprofloxacin, Cotrimoxazole, Gentamicin, Tobramycin |  |  | 1 |  |  |  |  |  |  |  |  |  |  |  |  |  |
| Augmentin, Ceftriaxone, Ciprofloxacin, Cotrimoxazole, Gentamicin, Tobramycin |  |  |  |  |  |  | 1 |  |  |  |  |  |  |  |  |  |
| Augmentin, Ceftriaxone, Ciprofloxacin, Cotrimoxazole, Gentamicin, Imipenem |  |  |  | 1 |  |  |  |  |  |  |  |  |  |  |  |  |
| Augmentin, Ceftriaxone, Ciprofloxacin, Cotrimoxazole, Gentamicin, Tetracycline |  |  |  | 5 |  |  |  |  |  |  |  |  |  |  |  |  |
| Augmentin, Ceftriaxone, Ciprofloxacin, Cotrimoxazole, Gentamicin, Tobramycin |  |  |  | 1 |  |  |  |  |  |  |  |  |  |  |  |  |
| Augmentin, Ceftriaxone, Ciprofloxacin, Cotrimoxazole, Tetracycline, Tobramycin |  | 1 | 1 | 1 |  |  |  |  |  |  |  |  |  |  |  |  |
| Augmentin, Ceftriaxone, Cotrimoxazole, Gentamicin, Tetracycline, Tobramycin |  | 2 |  | 2 |  |  |  |  |  |  |  |  |  |  |  |  |
| Augmentin, Ciprofloxacin, Cotrimoxazole, Gentamicin, Tetracycline, Tobramycin |  | 1 | 1 |  |  |  |  |  | 1 |  |  |  |  |  |  |  |
| Ceftazidime, Ceftriaxone, Chloramphenicol, Ciprofloxacin, Cotrimoxazole, Tetracycline |  |  |  | 1 |  |  |  |  |  |  |  |  |  |  |  |  |
| Ceftazidime, Ceftriaxone, Ciprofloxacin, Cotrimoxazole, Gentamicin, Tetracycline |  |  |  | 1 |  |  |  |  |  |  |  |  |  |  |  |  |
| Ceftazidime, Ceftriaxone, Ciprofloxacin, Cotrimoxazole, Gentamicin, Tobramycin | 3 |  |  |  |  |  |  |  |  |  |  |  |  |  |  |  |
| Ceftazidime, Ceftriaxone, Cotrimoxazole, Gentamicin, Tetracycline, Tobramycin | 1 |  |  |  |  |  |  |  |  |  |  |  |  |  |  |  |
| Ceftazidime, Ciprofloxacin, Cotrimoxazole, Gentamicin, Imipenem, Tetracycline | 1 |  |  |  |  |  |  |  |  |  |  |  |  |  |  |  |
| Ceftazidime, Ciprofloxacin, Cotrimoxazole, Gentamicin, Piperacillin-Tazobactam, Tetracycline | 1 |  |  |  |  |  |  |  |  |  |  |  |  |  |  |  |
| Ceftazidime, Ciprofloxacin, Cotrimoxazole, Gentamicin, Tetracycline, Tobramycin | 1 |  |  |  |  |  |  |  |  |  |  |  |  |  |  |  |
| Ceftriaxone, Ciprofloxacin, Cotrimoxazole, Gentamicin, Imipenem, Tobramycin | 1 |  |  |  |  |  |  |  |  |  |  |  |  |  |  |  |
| Ampicillin, Augmentin, Cefoxitin, Ceftazidime, Ceftriaxone, Nitrofurantoin, Tetracycline |  |  |  |  |  |  |  |  |  | 1 |  |  |  |  |  |  |
| Ampicillin, Augmentin, Ceftriaxone, Ciprofloxacin, Cotrimoxazole, Gentamicin, Imipenem |  |  |  |  |  | 1 |  |  |  |  |  |  |  |  |  |  |
| Ampicillin, Augmentin, Ceftazidime, Ceftriaxone, Ciprofloxacin, Tetracycline, Tobramycin |  |  | 1 |  |  |  |  |  |  |  |  |  |  |  |  |  |
| Ampicillin, Augmentin, Ceftriaxone, Ciprofloxacin, Cotrimoxazole, Gentamicin, Tobramycin |  |  |  | 12 | 1 |  |  |  |  |  |  |  |  |  |  |  |
| Ampicillin, Augmentin, Ceftriaxone, Ciprofloxacin, Cotrimoxazole, Tetracycline, Tobramycin |  |  | 1 |  |  |  |  |  |  |  |  |  |  |  |  |  |
| Ampicillin, Augmentin, Ceftriaxone, Cotrimoxazole, Gentamicin, Tetracycline, Tobramycin |  |  |  |  |  |  |  |  |  |  | 1 |  |  |  |  |  |
| Ampicillin, Ceftazidime, Ceftriaxone, Chloramphenicol, Cotrimoxazole, Gentamicin, Tobramycin |  | 1 |  |  |  |  |  |  |  |  |  |  |  |  |  |  |
| Ampicillin, Ceftazidime, Ceftriaxone, Ciprofloxacin, Cotrimoxazole, Gentamicin, Tobramycin |  |  |  | 1 |  |  |  |  |  |  |  |  |  |  |  |  |
| Ampicillin, Ceftriaxone, Chloramphenicol, Ciprofloxacin, Cotrimoxazole, Gentamicin, Tobramycin |  | 1 |  |  |  |  |  |  |  |  |  |  |  |  |  |  |
| Augmentin, Cefoxitin, Ceftazidime, Ceftriaxone, Ciprofloxacin, Cotrimoxazole, Gentamicin |  |  |  | 1 |  |  |  |  |  |  |  |  |  |  |  |  |
| Augmentin, Ceftazidime, Ceftriaxone, Ciprofloxacin, Cotrimoxazole, Gentamicin, Tetracycline | 1 |  | 1 | 1 |  |  |  |  |  |  |  |  |  |  |  |  |
| Augmentin, Ceftazidime, Ceftriaxone, Ciprofloxacin, Cotrimoxazole, Gentamicin, Tobramycin |  |  |  | 1 |  |  |  |  |  |  |  |  |  |  |  |  |
| Augmentin, Ceftazidime, Ceftriaxone, Ciprofloxacin, Cotrimoxazole, Nitrofurantoin, Tetracycline |  |  |  |  |  |  |  |  |  |  |  |  |  |  | 1 |  |
| Augmentin, Ceftazidime, Ceftriaxone, Ciprofloxacin, Cotrimoxazole, Imipenem, Tobramycin |  | 1 |  |  |  |  |  |  |  |  |  |  |  |  |  |  |
| Augmentin, Ceftazidime, Ceftriaxone, Ciprofloxacin, Cotrimoxazole, Tetracycline, Tobramycin |  |  | 4 | 1 |  |  |  |  |  |  |  |  |  |  |  |  |
| Augmentin, Ceftazidime, Ceftriaxone, Cotrimoxazole, Gentamicin, Tetracycline, Tobramycin |  |  |  | 3 |  |  | 2 |  |  |  |  |  |  |  |  |  |
| Augmentin, Ceftriaxone, Ciprofloxacin, Cotrimoxazole, Gentamicin, Imipenem, Penicillin |  | 1 |  |  |  |  |  |  |  |  |  |  |  |  |  |  |
| Augmentin, Ceftriaxone, Ciprofloxacin, Cotrimoxazole, Gentamicin, Nitrofurantoin, Tobramycin |  | 1 |  |  |  |  |  |  |  |  |  |  |  |  |  |  |
| Augmentin, Ceftriaxone, Ciprofloxacin, Cotrimoxazole, Gentamicin, Tetracycline, Tobramycin |  |  |  | 2 | 1 |  |  |  |  |  |  |  |  |  |  |  |
| Augmentin, Ceftriaxone, Ciprofloxacin, Cotrimoxazole, Nitrofurantoin, Tetracycline, Tobramycin |  |  |  | 1 |  |  |  |  |  |  |  |  |  |  |  |  |
| Ceftazidime, Ceftriaxone, Chloramphenicol, Ciprofloxacin, Cotrimoxazole, Gentamicin, Tobramycin |  | 1 |  |  |  |  |  |  |  |  |  |  |  |  |  |  |
| Ceftazidime, Ceftriaxone, Ciprofloxacin, Cotrimoxazole, Gentamicin, Meropenem, Tetracycline |  |  | 1 |  |  |  |  |  |  |  |  |  |  |  |  |  |
| Ceftazidime, Ceftriaxone, Ciprofloxacin, Cotrimoxazole, Gentamicin, Tetracycline, Tobramycin | 2 |  |  |  |  |  |  |  |  |  |  |  |  |  |  |  |
| Ceftriaxone, Chloramphenicol, Ciprofloxacin, Cotrimoxazole, Gentamicin, Tetracycline, Tobramycin |  |  |  |  |  | 1 |  |  |  |  |  |  |  |  |  |  |
| Ceftazidime, Chloramphenicol, Ciprofloxacin, Cotrimoxazole, Gentamicin, Imipenem, Tetracycline |  |  |  |  | 1 |  |  |  |  |  |  |  |  |  |  |  |
| Ceftriaxone, Cotrimoxazole, Gentamicin, Imipenem, Nitrofurantoin, Tetracycline, Tobramycin | 1 |  |  |  |  |  |  |  |  |  |  |  |  |  |  |  |
| Ampicillin, Augmentin, Ceftazidime, Ceftriaxone, Ciprofloxacin, Cotrimoxazole, Tetracycline, Tobramycin |  |  |  | 1 |  |  |  |  |  |  |  |  |  |  |  |  |
| Ampicillin, Augmentin, Ceftazidime, Ceftriaxone, Ciprofloxacin, Cotrimoxazole, Gentamicin, Tobramycin |  |  |  | 4 |  |  |  |  |  |  |  |  |  |  |  |  |
| Ampicillin, Augmentin, Ceftazidime, Ceftriaxone, Ciprofloxacin, Cotrimoxazole, Tetracycline, Tobramycin |  |  | 1 | 1 |  |  |  |  |  |  |  |  |  |  |  |  |
| Ampicillin, Augmentin, Ceftazidime, Ceftriaxone, Cotrimoxazole, Gentamicin, Tetracycline, Tobramycin |  |  |  | 1 |  |  |  |  |  |  |  |  |  |  |  |  |
| Ampicillin, Augmentin, Ceftriaxone, Chloramphenicol, Ciprofloxacin, Cotrimoxazole, Gentamicin, Imipenem |  | 1 |  |  |  |  |  |  |  |  |  |  |  |  |  |  |
| Ampicillin, Augmentin, Ceftriaxone, Chloramphenicol, Ciprofloxacin, Cotrimoxazole, Gentamicin, Tobramycin |  | 1 |  |  |  |  |  |  |  |  |  |  |  |  |  |  |
| Ampicillin, Augmentin, Ceftriaxone, Chloramphenicol, Cotrimoxazole, Gentamicin, Tetracycline, Tobramycin |  | 1 |  | 1 |  |  |  |  |  |  |  |  |  |  |  |  |
| Ampicillin, Augmentin, Ceftriaxone, Ciprofloxacin, Cotrimoxazole, Gentamicin, Imipenem, Tobramycin |  | 1 |  | 1 |  |  |  |  |  |  |  |  |  |  |  |  |
| Ampicillin, Augmentin, Ceftriaxone, Ciprofloxacin, Cotrimoxazole, Gentamicin, Nitrofurantoin, Tetracycline |  |  |  |  | 1 |  |  |  |  |  |  |  |  |  |  |  |
| Ampicillin, Augmentin, Ceftriaxone, Ciprofloxacin, Cotrimoxazole, Gentamicin, Nitrofurantoin, Tobramycin |  |  | 1 |  |  |  |  |  |  |  |  |  |  |  |  |  |
| Ampicillin, Augmentin, Ceftriaxone, Ciprofloxacin, Cotrimoxazole, Gentamicin, Tetracycline, Tobramycin |  | 2 | 2 | 4 |  |  |  |  |  |  |  |  |  |  |  |  |
| Ampicillin, Augmentin, Ceftriaxone, Ciprofloxacin, Cotrimoxazole, Tetracycline, Tobramycin |  |  | 1 |  |  |  |  |  |  |  |  |  |  |  |  |  |
| Ampicillin, Augmentin, Chloramphenicol, Ciprofloxacin, Cotrimoxazole, Gentamicin, Tetracycline, Tobramycin |  |  |  |  |  |  | 1 |  |  |  |  |  |  |  |  |  |
| Ampicillin, Ceftazidime, Ceftriaxone, Chloramphenicol, Ciprofloxacin, Cotrimoxazole, Gentamicin, Tetracycline |  |  | 1 |  |  |  |  |  |  |  |  |  |  |  |  |  |
| Augmentin, Ceftazidime, Ceftriaxone, Ciprofloxacin, Cotrimoxazole, Gentamicin, Tetracycline, Tobramycin |  | 2 |  | 5 |  |  |  |  |  |  |  |  |  |  |  |  |
| Augmentin, Ceftazidime, Ceftriaxone, Ciprofloxacin, Cotrimoxazole, Gentamicin, Imipenem, Tetracycline |  |  |  |  |  | 1 |  |  |  |  |  |  |  |  |  |  |
| Augmentin, Ceftazidime, Ceftriaxone, Ciprofloxacin, Gentamicin, Nitrofurantoin, Tetracycline, Tobramycin |  |  |  | 1 |  |  |  |  |  |  |  |  |  |  |  |  |
| Augmentin, Ceftriaxone, Ciprofloxacin, Cotrimoxazole, Gentamicin, Imipenem, Tetracycline, Tobramycin |  |  |  | 1 |  |  |  |  |  |  |  |  |  |  |  |  |
| Augmentin, Ceftriaxone, Ciprofloxacin, Cotrimoxazole, Gentamicin, Nitrofurantoin, Tetracycline, Tobramycin |  |  | 1 |  |  |  |  |  |  |  |  |  |  |  |  |  |
| Ceftazidime, Ceftriaxone, Chloramphenicol, Ciprofloxacin, Cotrimoxazole, Gentamicin, Imipenem, Tetracycline |  |  |  | 1 |  |  |  |  |  |  |  |  |  |  |  |  |
| Ceftazidime, Ceftriaxone, Ciprofloxacin, Cotrimoxazole, Gentamicin, Imipenem, Piperacillin-Tazobactam, Tobramycin | 1 |  |  |  |  |  |  |  |  |  |  |  |  |  |  |  |
| Ceftazidime, Ceftriaxone, Ciprofloxacin, Cotrimoxazole, Gentamicin, Piperacillin-Tazobactam, Tetracycline, Tobramycin | 1 |  |  |  |  |  |  |  |  |  |  |  |  |  |  |  |
| Ampicillin, Augmentin, Ceftazidime, Ceftriaxone, Chloramphenicol, Ciprofloxacin, Cotrimoxazole, Gentamicin, Tobramycin |  |  |  | 1 |  |  |  |  |  |  |  |  |  |  |  |  |
| Ampicillin, Augmentin, Ceftazidime, Ceftriaxone, Ciprofloxacin, Cotrimoxazole, Gentamicin, Nitrofurantoin, Tobramycin |  |  |  | 1 |  |  |  |  |  |  |  |  |  |  |  |  |
| Ampicillin, Augmentin, Ceftazidime, Ceftriaxone, Ciprofloxacin, Cotrimoxazole, Gentamicin, Tetracycline, Tobramycin |  |  | 2 | 1 |  |  |  |  |  |  |  |  |  |  |  |  |
| Ampicillin, Augmentin, Ceftazidime, Ceftriaxone, Ciprofloxacin, Cotrimoxazole, Nitrofurantoin, Tetracycline, Tobramycin |  |  | 1 |  |  |  |  |  |  |  |  |  |  |  |  |  |
| Ampicillin, Augmentin, Ceftriaxone, Ciprofloxacin, Cotrimoxazole, Gentamicin, Imipenem, Tetracycline, Tobramycin |  |  |  | 2 |  |  |  |  |  |  |  |  |  |  |  |  |
| Ampicillin, Augmentin, Ceftriaxone, Ciprofloxacin, Cotrimoxazole, Gentamicin, Imipenem, Tetracycline, Tobramycin |  |  |  | 1 |  |  |  |  |  |  |  |  |  |  |  |  |
| Augmentin, Ceftazidime, Ceftriaxone, Ciprofloxacin, Cotrimoxazole, Gentamicin, Imipenem, Tetracycline, Tobramycin | 1 |  |  |  |  |  |  |  |  |  |  |  |  |  |  |  |
| Augmentin, Ceftazidime, Ceftriaxone, Ciprofloxacin, Cotrimoxazole, Gentamicin, Nitrofurantoin, Tetracycline, Tobramycin |  |  | 1 |  |  |  |  |  |  |  |  |  |  |  |  |  |
| Ceftazidime, Ceftriaxone, Ciprofloxacin, Cotrimoxazole, Gentamicin, Imipenem, Nitrofurantoin, Tetracycline, Tobramycin | 1 |  |  |  |  |  |  |  |  |  |  |  |  |  |  |  |
| Ampicillin, Augmentin, Cefoxitin, Ceftazidime, Ceftriaxone, Ciprofloxacin, Cotrimoxazole, Gentamicin, Tetracycline, Tobramycin |  |  |  | 1 |  |  |  |  |  |  |  |  |  |  |  |  |
| Ampicillin, Augmentin, Ceftriaxone, Ciprofloxacin, Cotrimoxazole, Gentamicin, Imipenem, nitrofurantoin, Tetracycline, Tobramycin |  | 1 |  |  |  |  |  |  |  |  |  |  |  |  |  |  |
| Total | 40 | 37 | 86 | 133 | 16 | 13 | 17 | 1 | 2 | 6 | 4 | 15 | 1 | 1 | 5 | 1 |
