# Supplementary material for: A Six Years' Trend Analysis of Antimicrobial Resistance Among Bacterial Isolates at Public Health Institute in Amhara Region, Ethiopia
Source: Biomed Res Int. 2025 Jan 29;2025:7676973. doi: 10.1155/bmri/7676973 (PMC11824853; doi:10.1155/bmri/7676973)
Supplement: Supporting Information 4 — Table S4: Distribution of MDR gram-positive bacteria in APHI during the last 6 years, 2016–2021 (N = 195). [file 7676973.f4.docx]

Supplementary Table 4: Distribution of MDR gram-positive bacteria (2016-2021 ), at APHI, Northwest Ethiopia (N=195)

| antibiotics resistant to specific bacteria | CoNS Species | S aureus | Entrococcus Species | Staphylococcus species | Streptococcus species | Total |
| --- | --- | --- | --- | --- | --- | --- |
| Amoxicillin, Erythromycin |  | 1 |  |  | 1 | 2 |
| Ampicillin, Erythromycin |  |  | 2 |  |  | 2 |
| Ampicillin, Penicillin |  |  | 5 |  |  | 5 |
| Chloramphenicol, Erythromycin | 1 |  | 5 |  | 5 | 11 |
| Ciprofloxacin, Cotrimoxazole | 1 |  | 0 | 4 | 0 | 5 |
| Ciprofloxacin, Erythromycin | 2 | 1 |  |  |  | 3 |
| Ciprofloxacin, Gentamicin |  | 1 |  |  |  | 1 |
| Ciprofloxacin, Tetracycline |  | 1 | 1 |  |  | 2 |
| Clindamycin, Oxacillin | 1 |  |  |  |  | 1 |
| Cotrimoxazole, Erythromycin | 7 | 2 |  | 1 |  | 10 |
| Cotrimoxazole, Tetracycline | 2 | 1 |  |  |  | 3 |
| Erythromycin, Tetracycline |  | 2 |  |  |  | 2 |
| Erythromycin, Vancomycin |  |  | 2 |  |  | 2 |
| Oxacillin, Tetracycline | 1 |  |  |  |  | 1 |
| Ampicillin, Chloramphenicol, Erythromycin |  |  | 1 |  |  | 1 |
| Ampicillin, Erythromycin, Penicillin |  |  | 3 |  |  | 3 |
| Ampicillin, Erythromycin, Tetracycline |  |  | 1 |  |  | 1 |
| Azithromycin, Cotrimoxazole, Erythromycin | 2 | 1 |  |  |  | 3 |
| Cefoxitin, Ciprofloxacin, Cotrimoxazole | 1 |  |  |  |  | 1 |
| Cefoxitin, Ciprofloxacin, Tetracycline | 1 |  |  |  |  | 1 |
| Cefoxitin, Cotrimoxazole, Erythromycin | 1 |  |  |  |  | 1 |
| Chloramphenicol, Clindamycin, Cotrimoxazole | 1 |  |  |  |  | 1 |
| Chloramphenicol, Erythromycin, Tetracycline |  | 1 |  |  |  | 1 |
| Chloramphenicol, Gentamicin, Oxacillin | 1 |  |  |  |  | 1 |
| Chloramphenicol, Clindamycin, Erythromycin |  |  |  |  | 3 | 3 |
| Chloramphenicol, Erythromycin, Penicillin |  |  | 1 |  |  | 1 |
| Chloramphenicol, Erythromycin, Tobramycin |  |  | 1 |  |  | 1 |
| Ciprofloxacin, Cotrimoxazole, Erythromycin | 1 | 1 |  |  |  | 2 |
| Ciprofloxacin, Cotrimoxazole, Tetracycline | 1 |  |  |  |  | 1 |
| Ciprofloxacin, Clindamycin, Cotrimoxazole |  | 1 |  |  |  | 1 |
| Ciprofloxacin, Cotrimoxazole, Erythromycin | 2 |  |  |  |  | 2 |
| Ciprofloxacin, Cotrimoxazole, Gentamicin | 2 | 1 |  |  |  | 3 |
| Ciprofloxacin, Cotrimoxazole, Tetracycline | 1 | 1 |  |  |  | 2 |
| Ciprofloxacin, Erythromycin, Tetracycline |  |  | 2 |  |  | 2 |
| Clindamycin, Cotrimoxazole, Erythromycin | 3 |  |  |  |  | 3 |
| Clindamycin, Erythromycin, Gentamicin |  | 1 |  |  |  | 1 |
| Clindamycin, Erythromycin, Tetracycline |  | 1 |  |  |  | 1 |
| Cotrimoxazole, Erythromycin, Gentamicin | 1 | 1 |  |  |  | 2 |
| Cotrimoxazole, Erythromycin, Oxacillin | 1 |  |  |  |  | 1 |
| Cotrimoxazole, Erythromycin, Tetracycline | 1 | 1 |  | 2 | 1 | 5 |
| Cotrimoxazole, Gentamicin, Tetracycline | 1 |  |  |  |  | 1 |
| Cotrimoxazole, Oxacillin, Tetracycline | 1 |  |  |  |  | 1 |
| Amoxicillin, Ampicillin, Clindamycin, Cotrimoxazole |  |  | 1 |  |  | 1 |
| Ampicillin, Ciprofloxacin, Erythromycin, Tetracycline |  |  | 1 |  |  | 1 |
| Ampicillin, Erythromycin, Penicillin, Vancomycin |  |  | 2 |  |  | 2 |
| Cefoxitin, Chloramphenicol, Cotrimoxazole, Erythromycin |  | 1 |  |  |  | 1 |
| Chloramphenicol, Ciprofloxacin, Cotrimoxazole, Erythromycin | 1 |  |  |  |  | 1 |
| Chloramphenicol, Clindamycin, Cotrimoxazole, Erythromycin | 2 |  |  |  |  | 2 |
| Chloramphenicol, Cotrimoxazole, Erythromycin, Gentamicin | 1 |  |  |  |  | 1 |
| Chloramphenicol, Ciprofloxacin, Erythromycin, Tetracycline |  |  | 1 |  |  | 1 |
| Ciprofloxacin, Cotrimoxazole, Erythromycin, Gentamicin | 1 |  |  |  |  | 1 |
| Ciprofloxacin, Cotrimoxazole, Erythromycin, Gentamicin | 2 |  |  |  |  | 2 |
| Ciprofloxacin, Cotrimoxazole, Erythromycin, Oxacillin | 1 |  |  |  |  | 1 |
| Ciprofloxacin, Cotrimoxazole, Erythromycin, Tetracycline | 1 |  |  |  |  | 1 |
| Ciprofloxacin, Clindamycin, Cotrimoxazole, Erythromycin |  | 2 |  |  |  | 2 |
| Ciprofloxacin, Clindamycin, Erythromycin, Tetracycline |  | 1 |  |  |  | 1 |
| Ciprofloxacin, Cotrimoxazole, Erythromycin, Gentamicin | 2 | 2 |  |  |  | 4 |
| Ciprofloxacin, Cotrimoxazole, Erythromycin, Tetracycline | 4 | 1 |  |  |  | 5 |
| Ciprofloxacin, Cotrimoxazole, Gentamicin, Tetracycline | 3 | 1 |  |  |  | 4 |
| Ciprofloxacin, Gentamicin, Oxacillin, Tetracycline |  | 2 |  |  |  | 2 |
| Clindamycin, Cotrimoxazole, Erythromycin, Tetracycline | 1 |  |  |  |  | 1 |
| Cotrimoxazole, Erythromycin, Gentamicin, Tetracycline |  | 1 |  |  |  | 1 |
| Cotrimoxazole, Erythromycin, Oxacillin, Tetracycline | 1 |  |  |  |  | 1 |
| Cotrimoxazole, Erythromycin, Penicillin, Tetracycline | 1 |  |  |  |  | 1 |
| Ampicillin, Ceftriaxone, Ciprofloxacin, Cotrimoxazole, Nitrofurantoin |  |  | 1 |  |  | 1 |
| Cefoxitin, Chloramphenicol, Ciprofloxacin, Erythromycin, Tetracycline | 1 |  |  |  |  | 1 |
| Cefoxitin, Ciprofloxacin, Cotrimoxazole, Erythromycin, Tetracycline | 1 | 1 |  |  |  | 2 |
| Cefoxitin, Ciprofloxacin, Cotrimoxazole, Erythromycin, Gentamicin |  | 1 |  |  |  | 1 |
| Cefoxitin, Clindamycin, Cotrimoxazole, Erythromycin, Tetracycline | 1 |  |  |  |  | 1 |
| Chloramphenicol, Ciprofloxacin, Clindamycin, Cotrimoxazole, Erythromycin | 1 |  |  |  |  | 1 |
| Chloramphenicol, Ciprofloxacin, Clindamycin, Cotrimoxazole, Erythromycin | 1 |  |  |  |  | 1 |
| Chloramphenicol, Ciprofloxacin, Clindamycin, Erythromycin, Tetracycline | 1 |  |  |  |  | 1 |
| Chloramphenicol, Ciprofloxacin, Cotrimoxazole, Erythromycin, Oxacillin |  | 2 |  |  |  | 2 |
| Chloramphenicol, Clindamycin, Cotrimoxazole, Erythromycin, Oxacillin |  | 1 |  |  |  | 1 |
| Chloramphenicol, Clindamycin, Erythromycin, Oxacillin, Tetracycline | 1 |  |  |  |  | 1 |
| Chloramphenicol, Cotrimoxazole, Erythromycin, Gentamicin, Tetracycline | 1 |  |  |  |  | 1 |
| Chloramphenicol, Cotrimoxazole, Erythromycin, Oxacillin, Tetracycline | 1 |  |  |  |  | 1 |
| Ciprofloxacin, Clindamycin, Cotrimoxazole, Erythromycin, Gentamicin | 1 |  |  |  |  | 1 |
| Ciprofloxacin, Clindamycin, Cotrimoxazole, Erythromycin, Oxacillin | 1 |  |  |  |  | 1 |
| Ciprofloxacin, Cotrimoxazole, Erythromycin, Gentamicin, Oxacillin |  | 1 |  |  |  | 1 |
| Ciprofloxacin, Cotrimoxazole, Erythromycin, Gentamicin, Tetracycline | 1 |  |  |  |  | 1 |
| Ciprofloxacin, Clindamycin, Cotrimoxazole, Erythromycin, Gentamicin | 1 |  |  |  |  | 1 |
| Ciprofloxacin, Clindamycin, Cotrimoxazole, Erythromycin, Oxacillin |  | 1 |  |  |  | 1 |
| Ciprofloxacin, Clindamycin, Cotrimoxazole, Erythromycin, Tetracycline | 3 | 1 |  |  |  | 4 |
| Ciprofloxacin, Cotrimoxazole, Erythromycin, Gentamicin, Oxacillin | 2 |  |  |  |  | 2 |
| Ciprofloxacin, Cotrimoxazole, Erythromycin, Gentamicin, Tetracycline | 3 | 3 |  |  |  | 6 |
| Ciprofloxacin, Cotrimoxazole, Erythromycin, Oxacillin, Tetracycline | 1 |  |  |  |  | 1 |
| Ciprofloxacin, Cotrimoxazole, Erythromycin, Tetracycline, Tobramycin |  | 1 |  |  |  | 1 |
| Ciprofloxacin, Cotrimoxazole, Gentamicin, Oxacillin, Tetracycline | 1 |  |  |  |  | 1 |
| Clindamycin, Cotrimoxazole, Erythromycin, Oxacillin, Tetracycline | 2 |  |  |  |  | 2 |
| Ampicillin, Ciprofloxacin, Cotrimoxazole, Erythromycin, Gentamicin, Tetracycline |  | 1 |  |  |  | 1 |
| Chloramphenicol, Ciprofloxacin, Cotrimoxazole, Erythromycin, Oxacillin, Tetracycline | 1 |  |  |  |  | 1 |
| Chloramphenicol, Ciprofloxacin, Clindamycin, Cotrimoxazole, Erythromycin | 1 |  |  |  |  | 1 |
| Chloramphenicol, Ciprofloxacin, Clindamycin, Cotrimoxazole, Erythromycin, Gentamicin | 1 |  |  |  |  | 1 |
| Chloramphenicol, Ciprofloxacin, Clindamycin, Cotrimoxazole, Erythromycin, Tetracycline | 1 |  |  |  |  | 1 |
| Chloramphenicol, Clindamycin, Cotrimoxazole, Erythromycin, Gentamicin, Oxacillin | 1 |  |  |  |  | 1 |
| Ciprofloxacin, Cotrimoxazole, Erythromycin, Gentamicin, Oxacillin, Tetracycline |  | 1 |  |  |  | 1 |
| Ciprofloxacin, Clindamycin, Cotrimoxazole, Erythromycin, Gentamicin, Tetracycline | 3 |  |  |  |  | 3 |
| Ciprofloxacin, Cotrimoxazole, Erythromycin, Gentamicin, Oxacillin, Tetracycline | 4 | 1 |  |  |  | 5 |
| Azithromycin, Chloramphenicol, Ciprofloxacin, Clindamycin, Cotrimoxazole, Erythromycin, Tetracycline | 1 |  |  |  |  | 1 |
| Ceftriaxone, Chloramphenicol, Ciprofloxacin, Cotrimoxazole, Gentamicin, Tetracycline, Tobramycin |  |  | 1 |  |  | 1 |
| Chloramphenicol, Ciprofloxacin, Clindamycin, Cotrimoxazole, Erythromycin, Oxacillin, Tetracycline | 2 |  |  |  |  | 2 |
| Chloramphenicol, Ciprofloxacin, Cotrimoxazole, Erythromycin, Gentamicin, Oxacillin, Tetracycline |  | 1 |  |  |  | 1 |
| Ampicillin, Augmentin, Cefoxitin, Ceftriaxone, Chloramphenicol, Cotrimoxazole, Erythromycin, Gentamicin, Tetracycline | 1 |  |  |  |  | 1 |
| Total | 98 | 46 | 31 | 7 | 10 | 193 |
